# Targeted Delivery of α‐ketoglutarate to Macrophages in Bone: A Novel Therapeutic Strategy for Improving Fracture Healing in Type 2 Diabetes

**DOI:** 10.1002/advs.202415667

**Published:** 2025-02-25

**Authors:** Jing Wang, Jiahao Cao, Siqi Zhang, Hongli Chen, Xuebing Yu, Xinli Wang, Tianji Wang, Wei Cao, Wengang Dong, Xinsen Lin, Jia Li, Wei Lei, Yafei Feng

**Affiliations:** ^1^ Department of Orthopedics Xijing Hospital Fourth Military Medical University Xi'an Shaanxi 710032 China; ^2^ Department of Nuclear Medicine Xijing Hospital Fourth Military Medical University Xi'an Shaanxi 710032 China; ^3^ Key Laboratory of Aerospace Medicine of the Ministry of Education School of Aerospace Medicine Key Laboratory of Hazard Assessment and Control in Special Operational Environment of the Ministry of Education Fourth Military Medical University Xi'an Shaanxi 710032 China

**Keywords:** type 2 diabetes, macrophages, fracture healing, glutaminolysis, DNA methylation

## Abstract

Impaired fracture healing is a common complication in type 2 diabetes mellitus (T2DM), with limited effective treatments. This study investigates the role of macrophages in bone repair and introduces a novel therapeutic strategy. Reduced glutaminase (GLS) expression and glutaminolysis are found in macrophages from T2DM mice and monocytes from T2DM patients. Specific deletion of GLS in macrophages altered their phenotypes and delayed fracture healing in mice. Mechanistically, GLS deficiency reduced α‐ketoglutarate (α‐KG) levels in macrophages, which impairs bone morphogenetic protein 2 (BMP2) production by increasing cytosine methylation on the promoter, ultimately hindering osteogenic differentiation of bone marrow mesenchymal stem cells. Importantly, while systemic α‐KG supplementation deteriorates fracture healing in T2DM mice, a targeted delivery of α‐KG using α‐KG@Cy5.5@ALN‐Liposome to macrophages in bone markedly improves fracture healing. These findings underscore the critical role of macrophage glutaminolysis in fracture healing and propose targeted α‐KG delivery as a promising therapeutic intervention for improving fracture repair in T2DM patients.

## Graphical abstract

The study reveals that glutaminolysis in macrophages is inhibited under type 2 diabetes mellitus (T2DM) conditions, which impedes fracture healing by reducing bone morphogenetic protein 2 (BMP2) production through increased cytosine methylation on the promoter. The administration of alendronate‐modified bone‐targeting liposomes (α‐KG@Cy5.5@ALN‐Lipo), which deliver α‐ketoglutarate (α‐KG) to bone macrophages, significantly enhances fracture healing in a T2DM mouse model.

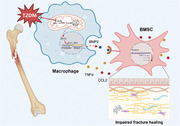

## 1 Introduction

In 2021, diabetes affected ≈537 million adults globally, with 90% of patients having type 2 diabetes mellitus (T2DM), according to the international diabetes federation (IDF), and the number is expected to rise to 643 million by 2030. T2DM is characterized by hyperglycemia and insulin resistance, which cause serious complications in the heart, kidneys, eyes, and feet.^[^ ^1^, ^2^ ^]^ Emerging evidence also points to its detrimental effects on skeletal health. Although patients with T2DM often have maintained bone mineral density (BMD), they face a significantly higher risk of fractures, particularly in the hip, vertebrae, and femur.^[^ ^3^, ^4^ ^]^ Furthermore, T2DM is associated with impaired fracture healing and a higher incidence of non‐unions compared to non‐diabetic individuals.^[^ ^5^, ^6^ ^]^ While anti‐diabetic drugs are widely used to manage fractures in T2DM patients, their primary effect is glucose level regulation, thus the effectiveness remains limited and even controversial.^[^ ^7^ ^]^ Clinical evidence identifies certain medications, including sulfonylureas, thiazolidinediones, and sodium‐glucose co‐transporter‐2 inhibitors (SGLT2‐i), may actually increase fracture risk.^[^ ^7^ ^,^ ^8^ ^]^ This underscores the urgent need to clarify the mechanisms behind impaired fracture healing in T2DM and develop more effective therapies.

Fracture healing is a complex process involving various cell types, including immune cells, bone marrow mesenchymal stem cells (BMSCs), and vascular endothelial cells.^[^ ^9^, ^10^ ^]^ As a key component of the bone microenvironment and one of the first immune cells to reach fracture sites, macrophages secret proinflammatory cytokines including TNFα and CCL2 to recruit BMSCs in the early phase.^[^ ^11^, ^12^ ^]^ As the healing proceeds, macrophages shift to a reparative phenotype, releasing prorepair cytokines such as TGF‐β and BMP‐2, which facilitate fracture healing.^[^ ^13^, ^14^ ^]^ However, T2DM exhibits a systemic chronic inflammation caused by the impaired transition of macrophages from a proinflammatory state to a reparative state, and the overactivation of inflammation and deficiency of prorepair cytokines secretion in macrophages impair tissue repair under T2DM.^[^ ^15^, ^16^ ^]^ Nevertheless, the precise mechanisms disrupting macrophage phenotype transition and function in T2DM have not been fully elucidated.

Metabolism has been reported closely associated with macrophage phenotype and function.^[^ ^17^, ^18^ ^]^ Glutamine, the most abundant non‐essential amino acid in plasma, is converted by glutaminase (GLS) in mitochondria into glutamate, which is further deaminated by glutamate dehydrogenase (GDH) into α‐ketoglutarate (α‐KG).^[^ ^19^ ^]^ α‐KG links glutaminolysis to the tricarboxylic acid (TCA) cycle and provides a key cofactor for ten‐eleven translocation hydroxylases (TETs), a kind of dioxygenase converts 5‐methyl‐cytosine (5mC) to 5‐hydroxy‐methyl‐cytosine (5hmC) in DNA and thus regulates chromatin accessibility and gene expression.^[^ ^20^, ^21^ ^]^ Furthermore, α‐KG controls inflammation and immune responses of macrophages in cancer and chronic inflammation‐related disease through TET enzymes mediated DNA demethylation.^[^ ^22^, ^23^, ^24^, ^25^ ^]^ Moreover, lower plasma levels of glutamine and a reduced glutamine‐to‐glutamate ratio are associated with a higher risk of T2DM,^[^ ^26^ ^]^ and glutamine supplementation has been shown to alleviate inflammation in obesity by reducing glycolysis in adipocytes and suppressing proinflammatory adipokine release, leading to improved insulin sensitivity and normalized glucose levels in T2DM.^[^ ^27^, ^28^ ^]^ However, the alterations of macrophage glutaminolysis under T2DM and its role in impaired fracture healing remain poorly understood.

In this study, we reveal that inhibited glutaminolysis in macrophages contributes to impaired fracture healing in T2DM, which can be rescued by targeted delivery of α‐KG to macrophages in bone. We first observed that macrophage glutaminolysis is inhibited under T2DM conditions, including high‐glucose and palmitic acid‐treated cells, T2DM mice, and T2DM patients. Additionally, GLS deletion in macrophages impaired fracture healing in vivo and hindered the osteogenic differentiation of BMSCs in vitro. Mechanistically, GLS deletion disrupted macrophage phenotype and increased proinflammatory cytokine secretion. Moreover, GLS deficiency reduced α‐KG levels, leading to decreased TET enzyme activity and increased DNA methylation at the *Bmp2* promoter, resulting in lower BMP2 production, and supplementation with α‐KG restored BMP2 secretion in GLS‐deficient macrophages. Finally, although systemic α‐KG administration worsened fracture healing due to increased mortality, targeted delivery of α‐KG to macrophages in bone reversed impaired healing in T2DM mice. These findings identify inhibited glutaminolysis in macrophages as a pivotal factor in impaired fracture healing and suggest that targeted α‐KG delivery presents a promising therapeutic approach for improving fracture healing in T2DM.

## 2 Results

### 2.1 Glutaminolysis is Inhibited in Macrophages of T2DM Mice and Patients

**Figure 1:**
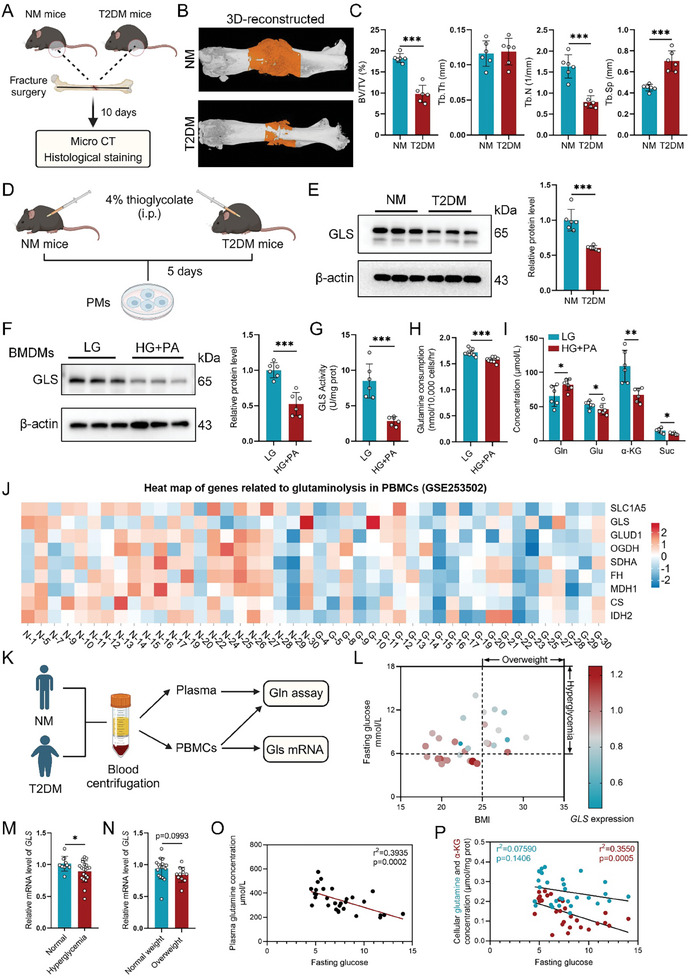
Glutaminolysis is inhibited in macrophages of T2DM mice and patients. A) Flow diagram illustrating the experimental design of fracture in NM and T2DM mice. B) Representative images and C) quantitative analysis of micro‐CT images demonstrating callus mineralization (*n* = 6). D) Experimental design for PMs isolation from NM and T2DM mice. E) Protein levels of GLS in PMs from T2DM and NM mice (*n* = 6). F) Protein levels of GLS in BMDMs treated with high glucose plus palmitic acid (HG+PA; 25 mm glucose plus 400 µm palmitic acid) or low glucose (LG; 5 mm glucose plus 20 mm mannitol) for 48 h (*n* = 6). G) GLS activity in BMDMs subjected to HG+PA or LG for 48 h (*n* = 6). H) Glutamine consumption in BMDMs treated with HG+PA or LG for 48 h (*n* = 9). I) Glutamine‐targeted metabolomics profiling showing levels of glutamine, glutamate, α‐KG, and succinate in BMDMs treated with HG+PA or LG for 24 h (*n* = 6). J) Heatmap derived from the GEO dataset (GSE253502), illustrating gene expression associated with glutamine metabolism in PBMCs from NM (N group) and T2DM (G group) patients (*n* = 22). K) Experimental design for monocyte isolation and subsequent assays from NM and T2DM patients (*n* = 10 in NM and *n* = 20 in T2DM). L) Bubble chart depicting BMI, fasting blood glucose levels, and *GLS* mRNA levels in monocytes from participants. M) Relative mRNA levels of *GLS* in monocytes from normoglycemic versus hyperglycemic individuals (*n* = 10 normoglycemic individuals and *n* = 20 hyperglycemic individuals). N) Relative mRNA levels of *GLS* in monocytes from normal weight versus overweight individuals (*n* = 19 normal weight individuals and *n* = 11 overweight individuals). O) Linear regression analysis of plasma glutamine levels versus fasting blood glucose levels in participants (*n* = 30). P) Linear regression analysis of cellular glutamine and α‐KG levels in monocytes versus fasting blood glucose levels in participants (*n* = 30). Data are shown as mean ± SD; *p < 0.05, **p < 0.01, and ***p < 0.001 by unpaired Student's two‐tailed t‐test.

Previous studies have demonstrated that fracture healing is impaired in patients with T2DM, often resulting in non‐union.^[^ ^29^ ^]^ To validate these findings in a murine model, we performed femur fracture surgery on a high‐fat diet (HFD)‐streptozotocin (STZ) induced T2DM mice and normal control (NM) mice (**Figure** **1**A; Figure S1A, Supporting Information). The T2DM model was successfully established, as evidenced by increased body weight, food intake, water consumption, and impaired glucose tolerance and insulin tolerance (Figure S1B–F, Supporting Information). At 10 days post‐fracture, T2DM mice exhibited reduced bone volume fraction (BV/TV) and trabecular number (Tb.N), alongside increased trabecular separation (Tb.Sp) compared to NM mice (Figure 1B,C). Furthermore, T2DM mice displayed smaller calluses with diminished cartilage content (Figure S2A,B, Supporting Information). These results confirm that fracture healing is impaired in T2DM mice.

Macrophages are crucial for fracture healing, and their function is influenced by glutamine metabolism.^[^ ^22^, ^30^ ^]^ To explore the effects of T2DM on macrophage glutaminolysis, we first isolated peritoneal macrophages (PMs) from NM and T2DM mice (Figure 1D). GLS protein levels were significantly lower in PMs from T2DM mice compared to NM mice (Figure 1E). Additionally, we isolated bone marrow‐derived macrophages (BMDMs) from 6‐week‐old C57BL/6 mice and treated cells with high glucose and palmitic acid (HG+PA; 25 mm glucose and 400 µm palmitic acid) for 48 h to imitate T2DM conditions in vitro.^[^ ^31^ ^]^ The results showed that HG+PA treatment significantly reduced GLS expression (Figure 1F). Moreover, GLS activity and glutamine consumption were markedly lower in HG+PA‐treated BMDMs compared to those treated with low glucose (LG) (Figure 1G,H). We also measured glutaminolysis metabolites, including glutamine, glutamate, and two TCA cycle intermediates, α‐KG and succinate, which are critical for macrophage function.^[^ ^22^, ^32^ ^]^ The results indicated an increase in glutamine levels following HG+PA exposure, while downstream metabolites, including glutamate, α‐KG, and succinate, were significantly decreased, confirming inhibited glutaminolysis in macrophages during T2DM (Figure 1I).

Furthermore, transcription profiling of peripheral blood mononuclear cells (PBMCs) from patients with T2DM and healthy controls (GSE253502) revealed a significant reduction in the expression of genes associated with glutamine metabolism in T2DM patients (Figure 1J). We then collected plasma and monocytes from T2DM patients and healthy controls (Figure 1K), and conducted a comprehensive analysis of body mass index (BMI), fasting blood glucose levels, and *GLS* mRNA levels in monocytes (Figure 1L). The results indicated that *GLS* expression in monocytes was significantly lower in individuals with hyperglycemia (fasting glucose > 6.0 mmol L^−1^) compared to those with normal blood glucose levels (fasting glucose < 6.0 mmol L^−1^, Figure 1M). Additionally, *GLS* expression was decreased in overweight participants (BMI > 25) relative to normal‐weight individuals (BMI < 25, Figure 1N, p = 0.0993). Linear regression analysis further demonstrated that plasma glutamine levels, along with cellular glutamine and α‐KG levels in monocytes, were negatively correlated with fasting blood glucose levels (Figure 1O,P). Together, these results suggest that macrophage glutaminolysis is inhibited in T2DM, primarily due to reduced expression and activity of GLS.

### 2.2 Specific Deletion of GLS in Macrophages Impairs Fracture Healing in Mice

**Figure 2:**
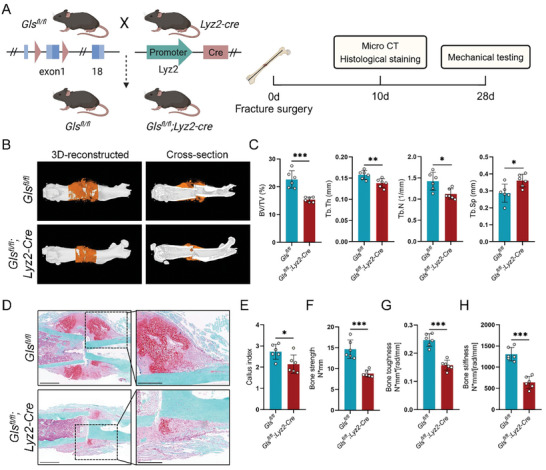
Specific deletion of GLS in macrophages impairs fracture healing in mice. A) Experimental design for *Gls^fl/fl^* and *Gls^fl/fl^;Lyz2‐Cre* mice to analyze fracture healing. B) Representative micro‐CT images and C) quantitative analysis showing callus mineralization 10 days post‐fracture in *Gls^fl/fl^* and *Gls^fl/fl^;Lyz2‐Cre* mice (*n* = 6). D) Representative Safranin O staining images of femur calluses in *Gls^fl/fl^* and *Gls^fl/fl^;Lyz2‐Cre* mice at 10 days post‐fracture (*n* = 6). Red indicates cartilage, and green indicates bone (Scale bars, 1 mm (left panels) and 0.5 mm (right panels)). E) Quantitative analysis of callus index in femurs of *Gls^fl/fl^* and *Gls^fl/fl^;Lyz2‐Cre* mice at 10 days post‐fracture (*n* = 6). F) Strength, G) toughness, and H) stiffness of callus at 28 days post‐fracture measured by three‐point bending tests (*n* = 6). Data are shown as mean ± SD; *p < 0.05, **p < 0.01, and ***p < 0.001 by unpaired Student's two‐tailed t‐test.

To explore the role of macrophage glutaminolysis in fracture healing, we generated *Gls^fl/fl^*;*Lyz2‐Cre* mice and assessed the fracture healing process in vivo (**Figure** **2**A). In BMDMs from *Gls^fl/fl^*;*Lyz2‐Cre* mice, we noted significant reductions in both mRNA and protein levels of GLS compared to *Gls^fl/fl^* controls (Figure S3A,B, Supporting Information). Micro‐CT imaging was used to evaluate mineralized callus formation, revealing that *Gls^fl/fl^*;*Lyz2‐Cre* mice had a smaller hard callus 10 days post‐fracture (Figure 2B). Quantitative analysis showed a decrease in BV/TV, Tb.Th, and Tb.N, while Tb.Sp was increased in *Gls^fl/fl^*;*Lyz2‐Cre* mice compared to *Gls^fl/fl^* controls (Figure 2C). Safranin O staining indicated a significant reduction in cartilage content within the callus of *Gls^fl/fl^*;*Lyz2‐Cre* mice (Figure 2D). We used the callus index, defined as the maximum callus diameter divided by the diameter of the adjacent diaphysis, to evaluate size differences between *Gls^fl/fl^*;*Lyz2‐Cre* and *Gls^fl/fl^* mice.^[^ ^33^ ^]^ Consistently, the callus index in *Gls^fl/fl^*;*Lyz2‐Cre* mice was smaller than that in *Gls^fl/fl^* controls (Figure 2E). Additionally, a three‐point bending test assessed the mechanical properties of the callus formed 28 days post‐fracture. Results showed that fractured femurs from *Gls^fl/fl^*;*Lyz2‐Cre* mice had reduced bone strength, stiffness, and toughness compared to those from *Gls^fl/fl^* mice (Figure 2F–H). Collectively, these findings underscore the critical role of macrophage glutaminolysis in fracture healing in mice.

### 2.3 GLS Deficiency in Macrophages Hinders Osteogenic Differentiation of BMSCs

**Figure 3:**
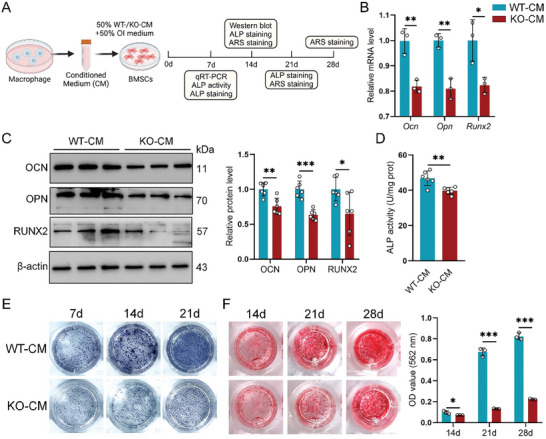
GLS deficiency in macrophages hinders the osteogenic differentiation of BMSCs. A) Schematic representation of BMDMs‐CM preparation and the experimental design for co‐culture and assays at various time points. B) mRNA levels of osteogenic markers (*Ocn*, *Opn*, and *Runx2*) in BMSCs co‐cultured with CM for 7 days, normalized to *β‐actin* (*n =* 3). C) Protein levels of osteogenic markers (OCN, OPN, and RUNX2) in BMSCs co‐cultured with CM for 28 days, normalized to β‐actin (*n =* 6). D) ALP activity measured in BMSCs co‐cultured with CM for 7 days (*n =* 6). E) Representative images of ALP staining in BMSCs co‐cultured with CM for 7, 14, and 21 days (*n =* 3). F) Representative images of Alizarin Red S staining and quantitative analysis of BMSCs co‐cultured with CM for 14, 21, and 28 days (*n =* 3). Data are shown as mean ± SD; *p < 0.05, **p < 0.01, and ***p < 0.001 by unpaired Student's two‐tailed t‐test.

Upon fracture healing, the osteogenic differentiation of BMSCs is regulated by cytokines released from macrophages.^[^ ^34^ ^]^ To assess the effects of GLS deletion in macrophages on the differentiation of BMSCs, we collected conditioned media (CM) from BMDMs isolated from *Gls^fl/fl^* and *Gls^fl/fl^;Lyz2‐Cre* mice, designated as WT‐CM and KO‐CM, respectively. CMs were then mixed with fresh osteogenic induction medium at a 1:1 ratio to culture BMSCs for evaluations (**Figure** **3**A). BMSCs treated with KO‐CM exhibited significantly lower mRNA and protein levels of osteogenic markers, including osteocalcin (OCN), osteopontin (OPN), and runt‐related transcription factor 2 (RUNX2) compared to those treated with WT‐CM (Figure 3B,C). Alkaline phosphatase (ALP) activity was also reduced in BMSCs exposed to KO‐CM compared to the WT‐CM group (Figure 3D). ALP staining confirmed fewer ALP‐positive cells in the KO‐CM group relative to the WT‐CM group (Figure 3E). Additionally, Alizarin Red S (ARS) staining revealed that KO‐CM inhibited calcium nodule formation, as evidenced by smaller sizes and fewer nodules in BMSCs treated with KO‐CM compared to the WT‐CM group, indicating impaired mineralization (Figure 3F). Collectively, these findings suggest that GLS deficiency in macrophages may alter the secretory profile, thereby hindering the osteogenic differentiation of BMSCs.

### 2.4 Aberrant Glutaminolysis Disrupts the Macrophages' Phenotype

**Figure 4:**
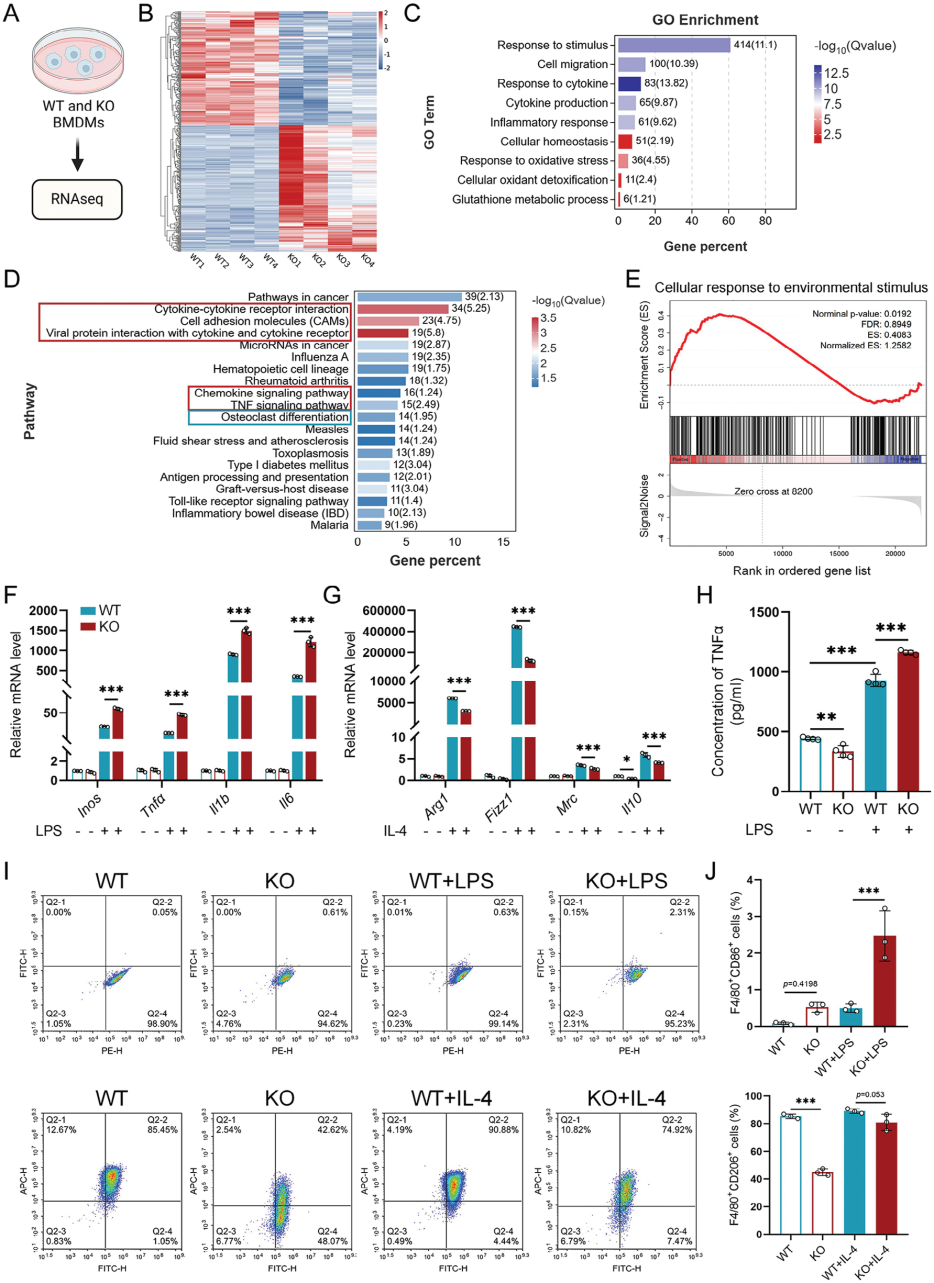
Aberrant glutaminolysis disrupts the macrophages' phenotype. A) Schematic overview of RNA‐seq analysis of BMDMs isolated from *Gls^fl/fl^ and Gls^fl/fl^;Lyz2‐Cre* mice (WT and KO BMDMs) (*n =* 4). B) Heatmap displaying differentially regulated genes in WT and KO BMDMs. C) GO enrichment analysis highlighting biological processes related to inflammation, cytokine production, and cellular homeostasis in WT and KO BMDMs. D) KEGG pathway enrichment analysis reveals the top 20 pathways enriched in WT and KO BMDMs. E) GSEA of genes involved in cellular responses to environmental stimulus. F) Pro‐inflammatory cytokines (*Inos, Tnfa, Il1b*, and *Il6*) mRNA expression in BMDMs stimulated with 100 ng mL^−1^ LPS for 6 h and G) anti‐inflammatory cytokines (*Arg1, Fizz1, Mrc*, and *Il10*) mRNA expression in BMDMs stimulated with 20 ng mL^−1^ IL‐4 for 24 h (*n =* 3). H) Concentrations of TNFα in the supernatants of WT and KO BMDMs treated with vehicle or LPS for 6 h (*n =* 4). I) Representative images and J) quantitative analysis showing the percentage of pro‐inflammatory macrophages (F4/80^+^CD86^+^) and anti‐inflammatory macrophages (F4/80^+^CD206^+^) in BMDMs stimulated with 100 ng mL^−1^ LPS for 6 h or 20 ng mL^−1^ IL‐4 for 24 h (*n =* 3). Data are shown as mean ± SD; **p < 0.01 and ***p < 0.001 by one‐way ANOVA.

Macrophages participate in all stages of fracture healing, displaying a variety of phenotypes and functions, which are closely tied to metabolic processes.^[^ ^17^ ^]^ To investigate how glutaminolysis influences macrophage phenotypes, we isolated BMDMs from *Gls^fl/fl^* and *Gls^fl/fl^;Lyz2‐Cre* mice and performed transcriptomic analysis via high‐throughput RNA sequencing (RNA‐Seq) (**Figure** **4**A). Differentially expressed genes (DEGs) were identified based on fold changes >1.5 and *p*‐values <0.05. The analysis showed that GLS deletion resulted in the downregulation of 195 genes and the upregulation of 217 genes (Figure 4B; Figure S4A,B, Supporting Information). Gene ontology (GO) enrichment analysis revealed significant changes in macrophage functions, including response to stimulus, cytokine production, and cellular homeostasis, between WT and KO macrophages (Figure 4C). Kyoto Encyclopedia of Genes and Genomes (KEGG) enrichment analysis highlighted significant enrichment of pathways associated with inflammation, including cytokines interactions, chemokine signaling, TNF signaling, and osteoclast differentiation among the top 20 enriched pathways (Figure 4D). Moreover, Gene Set Enrichment Analysis (GSEA) indicated an upregulation in the cellular response to environmental stimulus in KO macrophages compared to WT (Figure 4E). Notably, despite similar baseline levels, GLS deficiency led to increased mRNA levels of pro‐inflammatory markers (*Inos*, *Tnfα, Il1β*, and *Il6*) in response to lipopolysaccharide (LPS) stimulation compared to WT macrophages (Figure 4F). Conversely, mRNA levels of anti‐inflammatory markers (*Arg1*, *Fizz1, Mrc*, and *Il10*) were downregulated in KO macrophages upon interleukin‐4 (IL‐4) stimulation (Figure 4G). These results are consistent with findings from Liu et al., which indicated that glutamine deprivation disrupts macrophage phenotype.^[^ ^22^ ^]^ Correspondingly, levels of proinflammatory cytokines, including TNFα and CCL2, were increased in the supernatant of KO macrophages compared to WT under LPS stimulation (Figure 4H; Figure S4C, Supporting Information). Moreover, GLS deficiency led to an increase in the percentage of pro‐inflammatory (F4/80^+^CD86^+^) macrophages while simultaneously decreasing the percentage of anti‐inflammatory (F4/80^+^CD206^+^) macrophages (Figure 4I,J). In summary, these results indicate that aberrant glutaminolysis disrupts macrophage phenotype and triggers severe inflammation, potentially hindering fracture healing in the early stage.

### 2.5 GLS Deletion in Macrophages Reduces BMP2 Production Via Enhanced Cytosine Methylation on Promoter

**Figure 5:**
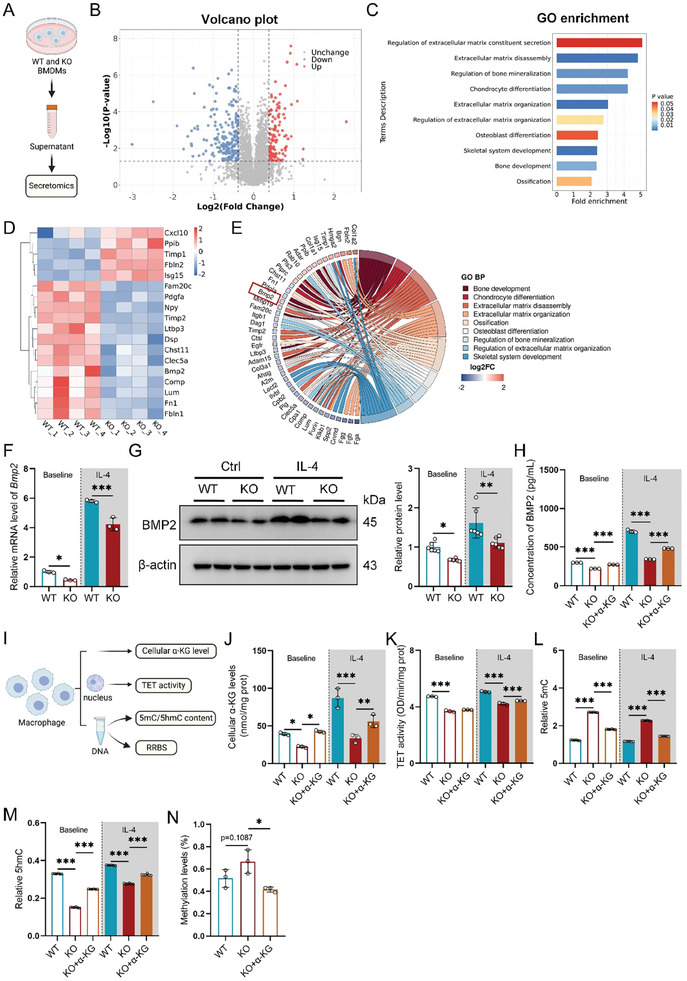
GLS deletion in macrophages reduces BMP2 production via enhanced cytosine methylation on promoter. A) Experimental design for analyzing the secretome of supernatants collected from WT and KO BMDMs (*n =* 4). B) Volcano plot illustrating differentially secreted proteins in the supernatants of WT and KO BMDMs. C) GO enrichment analysis of biological processes related to fracture healing in macrophages. D) Heatmap displaying differentially secreted proteins associated with osteoblast differentiation and proliferation in WT and KO BMDMs. E) Chord diagram showing the relationships between genes and biological processes relevant to fracture healing. F) mRNA levels of *Bmp2* in WT and KO BMDMs (*n =* 3). G) Protein levels of BMP2 in WT and KO BMDMs (*n =* 6). H) BMP2 concentrations in the supernatants of WT and KO BMDMs treated with vehicle or DM‐α‐KG, measured by ELISA (*n =* 3). I) Experimental design for DNA methylation assays in BMDMs. J) Cellular α‐KG levels in WT and KO BMDMs treated with vehicle or DM‐α‐KG (*n =* 3). K) TET activity levels in WT and KO BMDMs treated with vehicle or DM‐α‐KG (*n =* 3). L) Oxidative cytosine 5mC contents and M) reduced cytosine 5hmC contents in WT and KO BMDMs treated with vehicle or DM‐α‐KG (*n =* 6). N) Cytosine methylation levels in the promoter region of *Bmp2* in WT and KO BMDMs treated with vehicle or DM‐α‐KG (*n =* 3). Data are shown as mean ± SD; *p < 0.05, **p < 0.01, and ***p < 0.001 by one‐way ANOVA.

Macrophages are involved in fracture healing through cytokine secretion and interact with various cells including BMSCs.^[^ ^30^, ^35^ ^]^ To investigate the role of GLS in macrophage cytokine secretion, we then collected supernatants from WT and KO BMDMs to conduct secretomics analysis, which assessed differential cytokine secretion (**Figure** **5**A). Differentially secreted proteins were defined by fold changes >1.3 and *p*‐values <0.05. GLS deletion led to the upregulation of 123 cytokines and the downregulation of 200 cytokines (Figure 5B; Figure S5A,B, Supporting Information). KEGG enrichment showed that cytokine interaction including ECM‐receptor interaction and cytokine–cytokine receptor interaction was significantly altered in KO macrophages (Figure S5C, Supporting Information). In addition, the results revealed that the biological processes associated with these differentially secreted proteins involved bone mineralization, osteoblast differentiation, and ossification, all were critical to fracture healing (Figure 5C–E). These findings suggest that impaired glutaminolysis in macrophages disrupt fracture healing by altering cytokine secretion.

In the GO Chordal graph, bone morphogenetic protein 2 (BMP2), a crucial cytokine for fracture healing and a member of the TGF‐beta superfamily,^[^ ^36^ ^]^ was found to correlate with nearly all enriched biological processes related to fracture healing (Figure 5E). Then we evaluated BMP2 expression in BMDMs and observed that IL‐4 exposure increased BMP2 expression, while GLS deletion resulted in lower BMP2 mRNA and protein levels compared to WT macrophages (Figure 5F,G). Correspondingly, although BMP2 secretion was upregulated in BMDMs following IL‐4 treatment, KO BMDMs showed reduced BMP2 levels both at baseline and after IL‐4 exposure. Importantly, the addition of DM‐α‐KG restored BMP2 secretion in GLS‐deficient macrophages (Figure 5H).

To investigate how GLS deficiency affects BMP2 secretion, we focused on α‐KG dependent dioxygenases, particularly TET enzymes, which play a crucial role in DNA methylation and gene expression regulation (Figure 5I). Our prior results indicated that inhibited glutaminolysis in macrophages decreases α‐KG levels in T2DM, then we measured α‐KG levels in GLS‐deficient macrophages and observed a significant reduction of α‐KG in KO macrophages compared to WT group (Figure 5J). As a result, TET activity was diminished in KO macrophages due to lower α‐KG levels, which could be restored with DM‐α‐KG supplementation (Figure 5K). Additionally, GLS deletion increased global DNA 5‐methylcytosine (5‐mC) levels and decreased 5‐hydroxymethylcytosine (5‐hmC) levels in macrophages compared to the WT group, indicating enhanced cytosine methylation in DNA, which was reversed by DM‐α‐KG supplementation (Figure 5L,M).

For a comprehensive and quantitative analysis of DNA methylation, we utilized Reduced Representation Bisulfite Sequencing (RRBS), a technique designed for quantifying DNA methylation. Our results showed that the methylation level of the *Bmp2* promoter was elevated in KO BMDMs but decreased upon DM‐α‐KG treatment (Figure 5N). Together, these results confirm that GLS deficiency in macrophages inhibits BMP2 production by lowering α‐KG levels and inhibiting TETs activity, ultimately increasing cytosine methylation at the *Bmp2* promoter.

### 2.6 Targeted Delivery of α‐KG to Macrophages in Bone Rescues Fracture Healing of T2DM Mice

**Figure 6:**
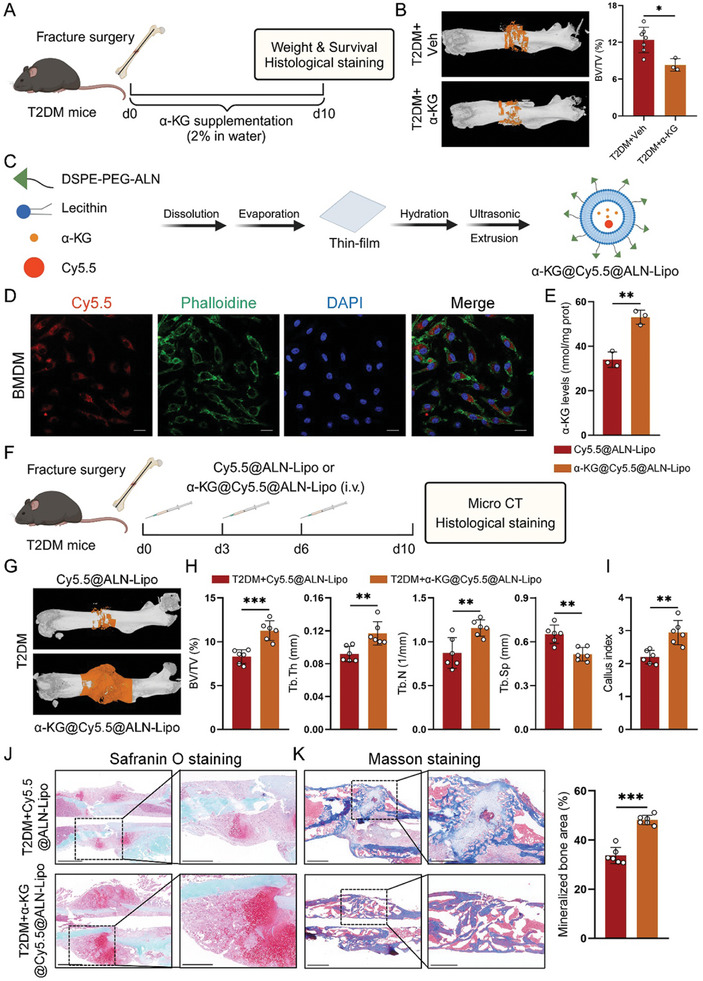
Targeted delivery of α‐KG to macrophages in bone rescues fracture healing of T2DM mice. A) Experimental design for oral α‐KG supplementation and subsequent analysis of fracture healing in T2DM mice. B) Representative micro‐CT images and quantitative analysis demonstrating callus mineralization at 10 days post‐fracture in T2DM mice treated with either α‐KG or vehicle (*n =* 7 for the T2DM + Vehicle group and *n =* 3 for the T2DM + α‐KG group). C) Schematic representation of a bone‐targeted liposome, decorated with alendronate (ALN), containing α‐KG and Cy5.5. D) Representative images of BMDMs treated with α‐KG@Cy5.5@ALN‐Lipo, indicating successful uptake of the liposome containing α‐KG and Cy5.5 (Scale bars, 20 µm). E) Cellular α‐KG levels in BMDMs treated with Cy5.5@ALN‐Lipo or α‐KG@Cy5.5@ALN‐Lipo (*n =* 3). F) Experimental design for administering Cy5.5@ALN‐Lipo or α‐KG@Cy5.5@ALN‐Lipo to T2DM mice, followed by the analysis of fracture healing. G) Representative micro‐CT images and H) quantitative analysis of callus formation at 10 days post‐fracture in T2DM mice treated with Cy5.5@ALN‐Lipo or α‐KG@Cy5.5@ALN‐Lipo (*n =* 6). I) Calculated callus index of bone in T2DM mice treated with Cy5.5@ALN‐Lipo or α‐KG@Cy5.5@ALN‐Lipo at 10 days post‐fracture (*n =* 6). J) Representative images of Safranin O staining in the callus of T2DM mice treated with Cy5.5@ALN‐Lipo or α‐KG@Cy5.5@ALN‐Lipo at 10 days post‐fracture, cartilage (red) and bone (green) (*n =* 6) (Scale bars, 1 mm (left panels) and 0.5 mm (right panels)). K) Representative images and quantitative analysis of Masson staining in the callus of T2DM mice treated with Cy5.5@ALN‐Lipo or α‐KG@Cy5.5@ALN‐Lipo at 28 days post‐fracture, mineralized bone (blue) and cartilage (transparent) (*n =* 6) (Scale bars, 1 mm (left panels) and 0.5 mm (right panels)). Data are shown as mean ± SD; *p < 0.05, **p < 0.01, and ***p < 0.001 by unpaired Student's two‐tailed t‐test.

To assess the effect of α‐KG on fracture healing in T2DM, we induced femur fractures in T2DM mice and administered α‐KG orally at a concentration of 2% (w/v) in water (**Figure** **6**A).^[^ ^37^ ^]^ Unexpectedly, α‐KG supplementation led to weakness, weight loss, and increased post‐fracture mortality (Figure S6A,B, Supporting Information).^[^ ^38^ ^]^ We subsequently collected femurs from the surviving T2DM mice and analyzed calluses using micro‐CT and Safranin O staining. The results revealed that systemic α‐KG supplementation impaired fracture healing by negatively affecting callus formation and mineralization (Figure 6B; Figure S6C–G, Supporting Information).

Given these results, we next explored the targeted delivery of α‐KG to macrophages in bone to evaluate its effects on fracture healing. We selected liposomes as the drug delivery system due to their capacity for phagocytosis by macrophages and the potential for enhanced targeting efficiency through modifications, such as the decorations of targeting peptides.^[^ ^39^ ^]^ Alendronate, known for its high affinity for bone, was chosen as the targeting peptide for our study.^[^ ^40^, ^41^ ^]^ We prepared bone‐targeted liposomes (α‐KG@Cy5.5@ALN‐Lipo) using the film‐rehydration method, combining DSPE‐PEG‐ALN, lecithin, α‐KG, and Cy5.5 as a labeling agent (Figure 6C). Transmission electron microscopy (TEM) confirmed the spherical morphology of the α‐KG@Cy5.5@ALN‐Lipo (Figure S7A, Supporting Information). Dynamic light scattering (DLS) analysis indicated that liposome sizes remained stable following α‐KG loading (diameters: 122.7 ± 3.35 nm vs 125.7 ± 1.64 nm; polydispersity index (PDI): 0.199 ± 0.004 vs 0.197 ± 0.008). Additionally, the zeta potential of α‐KG@Cy5.5@ALN‐Lipo was significantly lower than that of Cy5.5@ALN‐Lipo (−25.9 ± 1.65 mV vs −15.7 ± 0.94 mV) (Figure S7B–D, Supporting Information).

To assess the release profile of α‐KG from liposomes, we measured α‐KG concentrations in phosphate‐buffered saline (PBS) containing α‐KG@Cy5.5@ALN‐Lipo. Cumulative release data revealed that ≈60% of α‐KG was released within the first three days, while the remaining α‐KG was released gradually over the following four days (Figure S7E, Supporting Information). We evaluated the bone‐targeting efficiency of the liposomes using an in vivo imaging system (IVIS) to analyze the biodistribution of α‐KG@Cy5.5@ALN‐Lipo. Mice were sacrificed at 6 and 24 h post‐tail vein injection, and major organs, including the brain, heart, lungs, liver, spleen, kidneys, and hind limbs, were collected. The results indicated a significant accumulation of liposomes in the bone at 24 h (Figure S7F, Supporting Information). Additionally, Cy5.5 fluorescence signals were detected in macrophages incubated with α‐KG@Cy5.5@ALN‐Lipo, confirming successful uptake by macrophages (Figure 6D; Figure S7G, Supporting Information). Moreover, macrophages treated with α‐KG@Cy5.5@ALN‐Lipo exhibited significantly higher intracellular α‐KG levels compared to those treated with Cy5.5@ALN‐Lipo, indicating successful release of α‐KG within macrophages (Figure 6E).

To validate the effects of bone‐targeted liposomes on fracture healing, we administered either Cy5.5@ALN‐Lipo or α‐KG@Cy5.5@ALN‐Lipo to T2DM mice via tail vein injection after fracture surgery (Figure 6F). Micro‐CT analysis revealed that α‐KG@Cy5.5@ALN‐Lipo enhanced callus mineralization in T2DM mice at 10 days post‐surgery (Figure 6G). Quantitative analysis showed that the α‐KG@Cy5.5@ALN‐Lipo group had increased BV/TV, Tb.Th and Tb.N, along with a decrease in Tb.Sp compared to the Cy5.5@ALN‐Lipo group (Figure 6H). Furthermore, the callus index for femurs from α‐KG@Cy5.5@ALN‐Lipo‐treated T2DM mice was significantly greater than that of the Cy5.5@ALN‐Lipo group (Figure 6I). Safranin O staining further confirmed that α‐KG@Cy5.5@ALN‐Lipo increased callus size and cartilage content in T2DM mice compared to the Cy5.5@ALN‐Lipo group at 10 days post‐surgery (Figure 6J). Masson staining also revealed that α‐KG@Cy5.5@ALN‐Lipo enhanced mineralized bone formation at 28 days post‐surgery (Figure 6K). Moreover, compared to the Cy5.5@ALN‐Lipo group, the number of OCN^+^ osteoblasts increased while that of TRAP^+^ osteoclasts decreased in the callus of α‐KG@Cy5.5@ALN‐Lipo treated T2DM mice (Figure S8A–D, Supporting Information). In summary, our results suggest that bone‐targeted delivery of α‐KG effectively improves fracture healing in T2DM mice, primarily by ameliorating glutaminolysis and macrophage function through α‐KG supplementation.

## 3 Conclusion

This study elucidates critical insights into the metabolic dysfunctions underlying impaired fracture healing in T2DM. Our research identifies the suppression of GLS expression and the consequent reduction in glutamine‐derived α‐KG in macrophages as pivotal contributors to delayed fracture healing in T2DM. These findings establish a novel mechanistic link between the inhibition of glutaminolysis and osteogenic deficits, advancing our understanding of the metabolic underpinnings of this condition. Moreover, we have demonstrated that glutaminolysis significantly influences the inflammatory phenotype of macrophages and modulates the epigenetic landscape that governs BMP2 production. This mechanistic insight provides a fresh perspective on the role of macrophage metabolism in bone repair, highlighting its potential as a therapeutic target. In addition, our investigation into targeted α‐KG delivery to macrophages in bone offers a promising therapeutic strategy. By enhancing the specificity and efficacy of α‐KG treatment while mitigating the adverse effects associated with systemic delivery, this approach underscores the necessity of tailoring therapeutic interventions to the metabolic context specific to T2DM. Collectively, these findings not only deepen our understanding of the metabolic basis for impaired fracture healing in T2DM but also pave the way for the development of targeted interventions aimed at improving clinical outcomes.

Macrophages, as key responders to environmental changes, undergo metabolic reprogramming in response to stimuli.^[^ ^42^, ^43^ ^]^ In T2DM, characterized by dysregulated glucose and lipid metabolism, chronic inflammation persists due to excessive immune activation.^[^ ^44^, ^45^ ^]^ However, studies on amino acid metabolism, particularly glutamine metabolism, remain limited. Glutamine, the most abundant non‐essential amino acid in plasma, plays critical roles in tumor progression,^[^ ^46^ ^]^ stem cell differentiation,^[^ ^47^ ^]^ and aging‐related disorders.^[^ ^48^ ^]^ Clinical studies show an inverse relationship between the serum glutamine‐to‐glutamate ratio and diabetes risk,^[^ ^26^ ^]^ and glutamine supplementation has been shown to reduce inflammation in white adipose tissue.^[^ ^27^ ^]^ In this study, we demonstrated that GLS expression and activity, and glutaminolysis are inhibited in T2DM using monocytes from patients and healthy controls, PMs from T2DM and NM mice, and BMDMs exposed to high glucose and palmitic acid.

Macrophages are critical to all stages of fracture healing, interacting with immune cells, endothelial cells, and BMSCs through the secretion of cytokines. Among these, BMP2 is essential for promoting osteogenesis of BMSCs. In T2DM, however, chronic inflammation disrupts macrophage function, impairing the healing process.^[^ ^49^ ^]^ Although modulating macrophage phenotypes can improve bone repair,^[^ ^50^, ^51^ ^]^ the connection between macrophage glutaminolysis and fracture healing remains unclear. In our femur fracture model, we observed significantly compromised callus formation and mechanical strength in *Gls^fl/fl^;Lyz2‐Cre* mice compared to *Gls^fl/fl^* controls. In vitro, GLS‐deficient macrophages also inhibited osteogenic differentiation and mineralization of BMSCs.

RNA sequencing revealed that GLS deletion in macrophages exacerbated inflammation. This is consistent with previous findings that glutamine deficiency drives macrophages toward the pro‐inflammatory M1 phenotype, while α‐KG supplementation can reverse this effect.^[^ ^22^ ^]^ Mechanistically, oxidative stress, modulated by glutamine and α‐KG,^[^ ^22^, ^52^ ^]^ plays a role in inflammation. Glutathione (GSH), the primary intracellular antioxidant, relies on glutamate, a glutaminolysis product.^[^ ^53^ ^]^ GLS deficiency likely reduces GSH production and increases reactive oxygen species, leading to imbalanced macrophage phenotypes and impaired function. Moreover, Liu, P.S., et al. reported that glutamine‐derived α‐KG is vital for the activation of M2 macrophage via metabolic reprogramming (increased fatty acid oxidation) and Jmjd3‐dependent epigenetic modification of M2‐related genes.^[^ ^22^ ^]^ In our results, we observed that GLS deficiency led to decreased levels of α‐KG, which is likely the underlying reason for the altered macrophage phenotype observed in GLS knockout mice, contributing to the shift towards a pro‐inflammatory state.

Secretome analysis showed a marked reduction in BMP2 secretion following GLS ablation. BMP2, a member of the TGF‐β superfamily, regulates bone and cartilage formation through SMAD transcription factor activation.^[^ ^54^ ^]^ Macrophage polarization and metabolism influence BMP2 expression, and its secretion from M2 macrophages is crucial for bone repair.^[^ ^36^, ^55^ ^]^ Reduced BMP2 secretion in GLS‐deficient macrophages appears to be a key factor in delayed fracture healing in *Gls^fl/fl^;Lyz2‐Cre* mice. Epigenetic modifications control gene expression without changing the DNA sequence and are regulated by metabolites such as lactate, acetyl‐CoA, and α‐KG.^[^ ^56^ ^]^ α‐KG serves as a cofactor for TETs, a kind of dioxygenase, which influences gene expression by modulating DNA methylation.^[^ ^23^, ^57^ ^]^ In our study, glutaminolysis impairment due to T2DM or GLS deficiency reduced α‐KG levels in macrophages, inhibiting TET activity. This resulted in decreased levels of hydroxymethylated cytosine (5hmC) and increased methylated cytosine (5mC) in GLS‐deficient macrophages. To assess global DNA methylation, we performed genome‐wide analysis using RRBS. GLS deficiency in BMDMs increased methylation in the promoter region of *Bmp2*, a change reversed by α‐KG supplementation. These results suggest that glutaminolysis regulates macrophage cytokine secretion via DNA methylation, affecting bone development and regeneration.

Previous research has shown that α‐KG supplementation can reverse cellular dysfunction and pathological processes caused by glutamine deprivation.^[^ ^22^, ^47^ ^]^ Based on this, we administered α‐KG to T2DM mice to examine fracture healing. Despite reported benefits of α‐KG in T2DM, such as improving insulin sensitivity, enhancing cardiac mesenchymal cell survival, and promoting pericyte‐endothelial interactions,^[^ ^58^, ^59^ ^]^ our findings showed that oral α‐KG significantly impaired fracture healing in T2DM mice. Furthermore, α‐KG supplementation caused weight loss and increased mortality, likely due to enhanced gluconeogenesis, which raised glucose levels and worsened the metabolic state of the mice.^[^ ^38^ ^]^ To address these negative effects, we developed an alendronate‐decorated liposome for targeted delivery of α‐KG to macrophages in bone.^[^ ^40^ ^]^ Both in vivo and in vitro experiments confirmed its effective bone‐targeting properties and macrophage uptake. Micro‐CT and Safranin O staining showed that bone‐specific α‐KG delivery significantly improved fracture healing in T2DM mice.

In summary, this study elucidates the critical role of macrophage glutaminolysis in the fracture healing process, particularly within the context of T2DM. By targeting this metabolic pathway, we have demonstrated a promising therapeutic avenue that could significantly improve bone repair in diabetic patients, highlighting the potential of metabolic modulation as a therapeutic strategy in regenerative medicine.

## 4 Experimental Section

### Animals

All animal procedures were approved by the Animal Care and Use Committees at the Fourth Military Medical University (Xi'an, China) (Ethics Committee approval number: 20241481).

T2DM was induced in 4‐week‐old mice through an 8‐week high‐fat diet (HFD, 60%) (Research Diets, USA), followed by an intraperitoneal injection (i.p.) of STZ (40 mg kg^−1^ body weight) (Sigma Aldrich, Germany).^[^ ^49^ ^]^ The successful establishment of the T2DM model was confirmed by measuring fasting blood glucose (FBG) levels exceeding 11.1 mmol L^−1^, along with assessments of food intake, water consumption, body weight, intraperitoneal glucose tolerance tests (IPGTT) using glucose (2 g kg^−1^ body weight) after overnight fasting and insulin tolerance tests (ITT) using insulin (1 U kg^−1^ body weight, Novolin R) after 6 h of fasting.

*Gls^fl/fl^* and *Lyz2‐Cre* mice, both on a C57BL/6 background, were acquired from the Shanghai Model Organisms Center, Inc. (Shanghai, China). The *Gls^fl/fl^* mice were crossed with *Lyz2‐Cre* mice to achieve macrophage‐specific GLS deletion, resulting in the *Gls^fl/fl^*;*Lyz2‐Cre* genotype. Male mice aged eight to sixteen weeks were used in the studies, with littermates serving as controls.

All mice were housed under specific pathogen‐free conditions at 23 ± 2 °C and maintained on a 12‐h light‐dark cycle. Ad libitum access to food and water was provided for all animals.

### Surgical Procedures

To establish the femur fracture model, mice were anesthetized with pentobarbital sodium (0.5%) via i.p. The skin was sterilized using ethanol (75%), and a cross‐shaped incision was made at the knee to expose the patella, which was dislocated laterally to access the femoral condyles. A stainless‐steel intramedullary pin was inserted to stabilize the femur. Subsequently, a blunt dissection of the muscles was performed, and the femur was fractured at the midpoint with a dentist's microdrill. After the fracture, the muscles were repositioned, and the skin was sutured using a 6/0 nylon suture. Following the surgery, liposomes were injected intravenously into T2DM mice every three days (1.0 mg kg^−1^).

### Preparation of Bone‐Targeted Liposomes

Bone‐targeted liposomes were prepared via the film‐rehydration method.^[^ ^60^ ^]^ DSPE‐PEG2000‐ALN was procured from Qiyue Biology (Xi'an, China). A mixture containing DM‐α‐KG (Sigma, USA), Cy5.5, DSPE‐PEG‐ALN, and egg yolk lecithin was co‐dissolved in chloroform (3 mL) with a ratio of 5:1:5:50. This mixture was dried using a rotary evaporator under negative pressure at 50 °C to produce a thin film. The film was then hydrated with pre‐warmed (50 °C) double‐distilled water (3 mL) under vacuum and sonicated for 1 min (180 W, 5 s on, 5 s off) at room temperature. Liposomes were extruded through a 0.22 µm polycarbonate membrane (Millex, Ireland). Unloaded DM‐α‐KG and Cy5.5 were removed via dialysis using a polycarbonate membrane with a 30 nm pore size. The morphology of the liposomes was analyzed using TEM (FEI Talos L120C, USA). Furthermore, the liposome size, PDI, and zeta potential were assessed by dynamic light scattering (DLS) using a Zetasizer (Malvern Nano‐ZS, UK).

### Microcomputed Tomography

Fractured femurs from mice were collected, and the adjacent muscles were meticulously removed. Following fixation in 4% paraformaldehyde (PFA), the femurs were scanned with a SkyScan 1276 scanner (Bruker, Kartuizersweg, Belgium) at a spatial resolution of 8 µm, utilizing an X‐ray energy of 55 kVp and 250 µA. Three‐dimensional reconstruction of the bone was performed using CTVol software, and the resulting data were analyzed using CTAn.

### Histological Staining

Ten days after fracture, mice were sacrificed, and femurs were collected. The femurs were fixed in 4% PFA for 48 h and then decalcified in 15% EDTA‐Na_2_ (Sigma Aldrich) for 14 days. After decalcification, the femurs were embedded in paraffin and sectioned to a thickness of 5 µm. For Safranin O staining, Masson staining and TRAP staining, the paraffin sections were dewaxed and stained with Modified Safranin O and Fast Green Stain Kit (#G1371, Solarbio), Masson's Trichrome Stain Kit (#G1340, Solarbio) and Tartrate‐Resistant Acid Phosphatase(TRAP) Stain Kit (#G1492, Solarbio), respectively, according to the manufacturer's instructions. For immunohistochemical staining of osteoblasts, the sections were blocked with 1% BSA at room temperature for 1 h and then incubated with osteocalcin (#20277‐1‐AP, Proteintech) primary antibody at 4 °C overnight. Then the slides were incubated with an HRP‐Goat anti‐rabbit recombinant secondary antibody (RGAR011, Proteintech) at room temperature for 1 h. The antibody‐antigen complex was visualized by DAB Horseradish Peroxidase Color Development Kit (P0203, Beyotime). The sections were dehydrated and mounted with neutral resin for microscopic observation (Olympus, Tokyo, Japan). Then the statistics of images were performed using Image‐J.

### Biomechanical Assessments

To assess the biomechanical properties of fractured femurs, a three‐point bending test was performed using a mechanical testing machine (WDW‐100; Shanghai Bairoe Instruments Co., Ltd., Shanghai, China). The femurs were placed on two supporting rods and loaded until fracture at a displacement rate of 5 mm min^−1^. Bone strength, toughness, and stiffness were determined from the load‐deformation curves.

### Human Studies

Human studies were reviewed and approved by the Medical Ethics Committee of the First Affiliated Hospital of the Fourth Military Medical University (Ethics Committee approval number: KY20213177‐1). Participants were informed about the research project and sample collection procedures, and informed consent was obtained.

Patients diagnosed with T2DM were defined as those with fasting blood glucose levels of ≥ 7.0 mmol L^−1^ or HbA1c levels > 6.5%. Healthy controls were defined by fasting blood glucose levels of ≤ 6.0 mmol L^−1^.^[^ ^61^ ^]^ Exclusion criteria comprised a history of heavy alcohol use, pregnancy or lactation in female participants, diagnoses of autoimmune diseases, infectious diseases, hematological disorders, or cancers within the past 6 months, severe trauma or surgery within the past three months, and diagnoses of hepatitis, liver cirrhosis, or liver cancer. Individual characteristics are summarized in Table S1 (Supporting Information).

Blood samples ranging from six to eight milliliters were collected from eligible patients. PBMCs were isolated using Ficoll‐Paque PLUS (17144002, Cytiva, China) via density gradient centrifugation. Following centrifugation, serum was collected from the supernatant for further analysis, and PBMCs were purified using PBS. Monocytes were positively selected from whole PBMCs using antiCD14^+^ microbeads (Miltenyi Biotec) for the following experiments.^[^ ^62^ ^]^

### Cell Culture

All cells were cultured in a 37 °C incubator with 5% CO_2_, with culture media supplemented with penicillin (1000 U mL^−1^) and streptomycin (100 mg mL^−1^).

Primary BMDMs were isolated from 6‐ to 8‐week‐old mice as previously described.^[^ ^63^ ^]^ Bone marrow was flushed from the femur and tibias using PBS. After lysing red blood cells, the remaining cells were cultured in BMDM medium, consisting of DMEM (HyClone, USA) supplemented with 10% FBS (InCellGene, Germany), 15% L929 conditioned medium, and 1% penicillin‐streptomycin (Invitrogen, USA). After a 7‐day incubation, BMDMs were digested with Accutase (#PC‐90011, PlantChemMed) for subsequent experiments. To induce a pro‐inflammatory phenotype (M1 polarization), BMDMs were treated with LPS (100 ng mL^−1^) (Peprotech, USA) for 6 h. For the induction of an anti‐inflammatory phenotype (M2 polarization), BMDMs were treated with IL‐4 (20 ng mL^−1^) (Peprotech) for 24 h.

Primary BMSCs were isolated from 4‐week‐old C57BL/6 mice using a procedure as previously described.^[^ ^64^ ^]^ The medium was adjusted to BMSC medium, consisting of α‐MEM (HyClone) supplemented with 15% FBS and 1% penicillin‐streptomycin. To induce osteoblastic differentiation of BMSCs for ALP activity assays or Alizarin Red S staining, the cells were allowed to reach 90% confluency. The BMSC medium was then replaced with osteogenic induction medium, which included α‐MEM supplemented with 10% FBS, β‐glycerol phosphate (10 mm) (Sigma Aldrich), ascorbic acid (50 µg mL^−1^) (Sigma Aldrich), and dexamethasone (10^−8^ m) (Sigma Aldrich) for the specified duration.

### qRT‐PCR

The total RNA of cells was isolated using the Trizol reagent (Sigma) following the manufacturer's instruction, and then reverse transcribed into cDNA using the Hifair II 1st Strand cDNA Synthesis SuperMix (#11120ES60, Yeasen, Shanghai, China). Next, qRT‐PCR was performed using Hieff qPCR SYBR Green Master Mix (#11201ES08, Yeasen) on a BioRad Real‐time PCR System (CFX96; BioRad, Hercules, CA, USA). The primer sequences are listed in Table S2 (Supporting Information). All data were normalized to *36B4* (BMDM) or *β‐actin* (BMSC) expression. Relative mRNA levels of dividual genes were calculated by 2^−△△CT^ method.

### Flow Cytometry

BMDMs were digested with Accutase (#PC‐90011, PlantChemMed) and rinsed with PBS. Then the cell suspensions were washed twice with PBS, and stained with PE‐F4/80 (#PE‐98236, Proteintech) and FITC‐CD86 (#FITC‐65068, Proteintech) or FITC‐F4/80 (#123107, BioLegend) and APC‐CD206 (#141707, BioLegend). Cells were then incubated for 15 min at room temperature in the dark, and analyzed by flow cytometry (LSRFortessa; BD, Franklin Lakes, NJ, USA). The results were analyzed by FlowJo 7.6.5 software (Tree Star Inc., Ashland, OR, USA).

### Western Blot

Western blot was carried out as previously described.^[^ ^51^ ^]^ Cells were lysed and protein concentration were quantified through a BCA protein assay kit (#23225, Thermo Fisher). Further, equal amounts of proteins were separated by SDS‐PAGE gel electrophoresis and transferred onto a polyvinylidene fluoride (PVDF) membrane. After blocked with 5% skimmed milk at room temperature for 1 h, the membrane was incubated with primary antibodies against GLS (#12855‐1‐AP, Proteintech), Osteopontin (#25715‐1‐AP, Proteintech), Osteocalcin (#20277‐1‐AP, Proteintech), RUNX2 (#20700‐1‐AP, Proteintech), BMP2 (#66383‐1‐Ig, Proteintech), and β‐actin (AP0060, Bioworld Technology) as loading control at 4 °C overnight. Then the blots were incubated with an HRP‐conjugated secondary antibody (1:5000 dilution, Santa Cruz Biotech) for 1 h at room temperature. Protein levels were semi‐quantified by scanning densitometry and analyzed with Image J software.

### ELISA

The supernatants of BMDMs were collected, and centrifuged at 12 000 rpm for 30 min to remove the debris of cells. The concentrations of cytokines were measured with ELISA kit: CCL2 (#SEA087Mu, Cloud‐clone), TNF‐α (#SEA133Mu, Cloud‐clone), and BMP2 (#SEA013Mu, Cloud‐clone) following the manufacturers’ instructions.

### Glutamine and α‐KG Measurements

Plasma and cellular glutamine or α‐KG were detected using a Glutamine (Gln) Content Assay Kit (#BC5300, Solarbio) or Amplite Colorimetric α‐Ketoglutarate Quantitation Kit (#10085, AAT Bioquest) separately according to the manufacturer's instructions. Briefly, BMDMs were lysed and the extracts or plasma were added to the working solution and incubated at 37 °C for 1 h in the dark. The absorbance was measured at a wavelength of 450 nm (glutamine) or 570 nm (α‐KG). Glutamine and α‐KG in samples were calculated using the standard curve and equation.

### Measurements of Enzymatic Activity

Glutaminase activity was determined using a GLS Assay Kit (#BC1450, Solarbio) according to the manufacturer's instructions. In brief, BMDMs were lysed, and the extracts were added to the working solution and incubated at 37 °C for 1 h. The absorbance was measured at a wavelength of 630 nm. A unit of GLS activity was defined as 1 mg protein catalyzing glutamine to generate 1 µmol ammonia per hour at 37 °C. The data were expressed as U mg^−1^ protein.

TET activity was determined using an EpiQuik Nuclear Extraction Kit I (#OP‐0002, EpigenTek) and Epigenase 5mC Hydroxylase TET Activity/Inhibition Assay Kit (#P‐3086, EpigenTek) according to the manufacturer's instructions.^[^ ^23^ ^]^ Briefly, to prepare nuclear extracts, BMDMs were washed with PBS twice and scraped cells into a 15 mL conical tube. Add the extraction Buffer and incubate on ice for 10 minutes, then centrifuge the preparation for 1 min at 12 000 rpm, and carefully remove the cytoplasmic extract from the nuclear pellet. To detect TET activity, working solutions were prepared as instructed and described. Four microliters of nuclear extracts were added to the working solution and incubated at 37 °C for 90 min. Finally, the absorbance was read on a microplate reader within 2 to 10 min at 450 nm with an optional reference wavelength of 655 nm. TET activity was calculated using the formulas in the instructions.

### 5mC and 5hmC Detection

DNA was isolated using FitAmp Blood and Cultured Cell DNA Extraction Kit (#P‐1018, EpigenTek). Levels of 5‐mC and 5‐hmC were determined by MethylFlash Global DNA Methylation (5‐mC) ELISA Easy Kit (Colorimetric) (#P‐1030, EpigenTek) and MethylFlash Global DNA Hydroxymethylation (5‐hmC) ELISA Easy Kit (Colorimetric) (#P‐1032, EpigenTek). All steps were performed according to the manufacturer's instructions. Briefly, 100 ng DNA was used, and an absorbance at 405 nm was recorded using the microplate reader.

### The α‐KG Release of Bone‐Targeted Liposome

Release of α‐KG from liposomes was investigated by placing 1 mL of α‐KG@Cy5.5@ALN‐Lipo in dialysis bags (MWCO = 3 500 Da, Thermo, USA) as previously reported.^[^ ^60^ ^]^ The dialysis bags were incubated in sterile PBS (40 mL) incubated at 37 °C with agitation at 100 rpm. At the scheduled time points, 1 mL of PBS was taken out and replaced by the same volume of fresh PBS. The released medium was vortexed and analyzed by Amplite Colorimetric α‐Ketoglutarate Quantitation Kit (#10085, AAT Bioquest), the detailed method was mentioned above. The tests were conducted in triplicate, and the average data were calculated.

### Biodistribution of Bone‐Targeted Liposome

For biodistribution analysis, liposomes were i.v. injected into 8‐week‐old C57BL6 mice (1.0 mg kg^−1^ body weight);^[^ ^65^ ^]^ the kidneys, spleen, lungs, brain, heart, liver, and hind limbs were dissected 6 or 24 h post‐injection and were subjected to biophotonic imaging assay for evaluation of organ distribution of liposomes using the IVIS Spectrum In Vivo Imaging System (PerkinElmer).

### Alkaline Phosphatase (ALP) Activity Assay And Alizarin Red Staining

To analyze the osteogenic differentiation of BMSC, cells were induced for designated days with an osteogenic induction medium mixed with macrophage conditioned medium. ALP activity was detected using an Alkaline Phosphatase Assay Kit (#P0321S, Beyotime) according to the manufacturer's instructions. To perform staining, BMSCs were washed three times with PBS and fixed with 4% paraformaldehyde for 20 min at RT. ALP staining was performed with BCIP/NBT Alkaline Phosphatase Colorpment Kit (#C3206, Beyotime), and incubated in the working solution for 30 min at room temperature. Alizarin Red S staining was stained with Alizarin Red S solution (#C0138, Beyotime) for 15 min. Then the BMSCs were washed with deionized water and visualized using microscopy. The calcium nodules were resolved with 10% cetylpyridinium chloride and the absorbance was detected at 562 nm.

### RNA Sequencing

Total RNA was extracted from BMDMs using Trizol reagent (Sigma), and rRNA was subsequently removed. mRNA with a poly‐A tail was enriched from each sample using oligo (dT) magnetic beads and fragmented into smaller pieces through ultrasonic treatment. The integrity and purity of the RNA were assessed. The first strand of cDNA was synthesized with the M‐MuLV reverse transcriptase system, using the RNA fragments as templates and random oligonucleotides as primers. The second strand of cDNA was synthesized in a reaction containing dNTPs and DNA polymerase I. Double‐stranded cDNA was purified and modified using AMPure XP beads to isolate fragments of ≈200 bps. cDNA amplification was performed, and the products were purified again with AMPure XP beads to create the library. For functional enrichment analysis, GO term analysis was conducted, categorizing the data into three subgroups: biological process (BP), cellular component (CC), and molecular function (MF). Additionally, the KEGG database was used to identify significantly enriched KEGG pathways at a threshold of *p* < 0.05.

### Secretomics Analysis

BMDMs were seeded at a density of 5.5 × 10^6^ cells in 10 cm culture dishes for 24 h, followed by a 48‐h incubation in serum‐free DMEM to collect the supernatant. Secretome profiling was conducted by Jingjie PTM BioLab Co. Ltd (Hangzhou, Zhejiang, China). Cellular debris was removed from the serum samples by centrifugation at 12 000 g for 10 min at 4 °C. The supernatant was subsequently transferred to a new centrifuge tube for concentration. For digestion, trypsin was added at a 1:50 trypsin‐to‐protein mass ratio for overnight digestion. The tryptic peptides were dissolved in solvent A (0.1% formic acid, 2% acetonitrile in water) and separated using a gradient of solvent B (0.1% formic acid in 90% acetonitrile) over 60 min. The separated peptides were analyzed with an Orbitrap Exploris 480 mass spectrometer (ThermoFisher Scientific), with the automatic gain control (AGC) target set at 3E6 and the maximum injection time set to Auto. The resulting MS/MS data were processed using the MaxQuant search engine (v. 1.6.15.0) and analyzed against the UniProt Mus musculus database. Bioinformatics analysis was performed utilizing OmicStudio tools.

### Statistical Analysis

The number of independent experiments and the sample size (*n*) for each experiment were detailed in the figure legends. Statistical analysis was performed using GraphPad Prism v8.0 (GraphPad, La Jolla, CA, USA). All data were presented as mean ± SD. The methods of statistic analysis of significant differences between groups were described in figure legends. *p* ≤ 0.05 was considered statistically significant.

## Conflict of Interest

The authors declare no conflict of interest.

## Author Contributions

J.W., J.C., and S.Z. contributed equally to this work as co‐first authors. J.W., Y.F., and J.L. conceptualized the study. J.W., J.C., X.Y., X.W., W.C., W.D., T.W., and X.L. performed in vivo experiments including femur fracture surgery or pharmacological treatment. J.W., J.C., X.W., and W.D. performed in vitro experiments. J.W., J.C., S.Z., and H.C. performed RNA‐seq, secretomes, and RRBS data analysis. J.W., J.C., S.Z., and T.W. analyzed the data. J.W., J.C., and S.Z. visualized the data. Y.F., W.L., and J.L. acquired funding to support the project. J.W., S.Z., and H.C. wrote the original draft of the manuscript. Y.F., W.L., and J.L. reviewed and edited the manuscript.

[Correction added on 02 May 2025, after first online publication: Author Contributions statement has been added.]

## Supporting information



Supporting Information

Supplemental Table 1

## Data Availability

The data that support the findings of this study are available in the supplementary material of this article.
