# Supplementary material for: Targeted Delivery of α‐ketoglutarate to Macrophages in Bone: A Novel Therapeutic Strategy for Improving Fracture Healing in Type 2 Diabetes
Source: Adv Sci (Weinh). 2025 Feb 25;12(23):2415667. doi: 10.1002/advs.202415667 (PMC12199480; doi:10.1002/advs.202415667)
Supplement: Supplementary file 1 — Supporting Information [file ADVS-12-2415667-s001.docx]

Supporting Information

Title

Targeted Delivery of α-ketoglutarate to Macrophages in Bone: a Novel Therapeutic Strategy for Improving Fracture Healing in Type 2 Diabetes

*Jing Wang, Jiahao Cao, Siqi Zhang, Hongli Chen, Xuebing Yu, Xinli Wang, Tianji Wang, Wei Cao, Wengang Dong, Xinsen Lin, Jia Li*, Wei Lei*, Yafei Feng**

**Supplementary Figures**

**
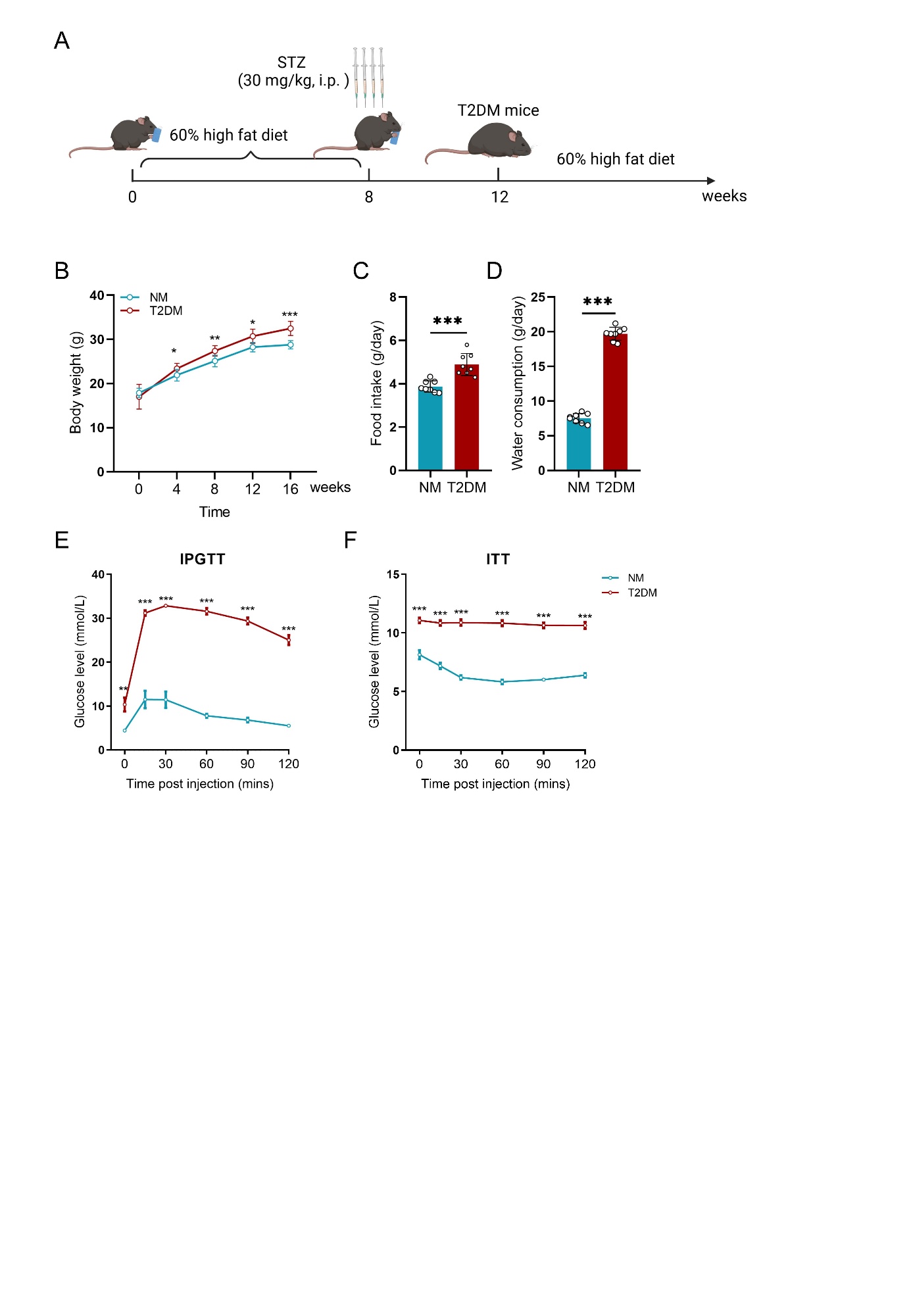
**

**Figure S1.** Type 2 diabetes mellitus (T2DM) mice model was induced with high fat diet (HFD) and streptozotocin (STZ) injection.A) Experimental design showing establishment of HFD/STZ induced T2DM mice model. B) Body weight, C) food intake and D) water consumption of T2DM and normal (NM) mice at 4 weeks after STZ injection (*n* = 8). E) Blood glucose levels during Intraperitoneal glucose tolerance test (IPGTT) and F) Insulin tolerance test (ITT) at 4 weeks after STZ injection (*n* = 8). Data are shown as mean ± SD; **p* < 0.01, ***p* < 0.01 and ****p* < 0.001 by unpaired Student’s two-tailed t-test.


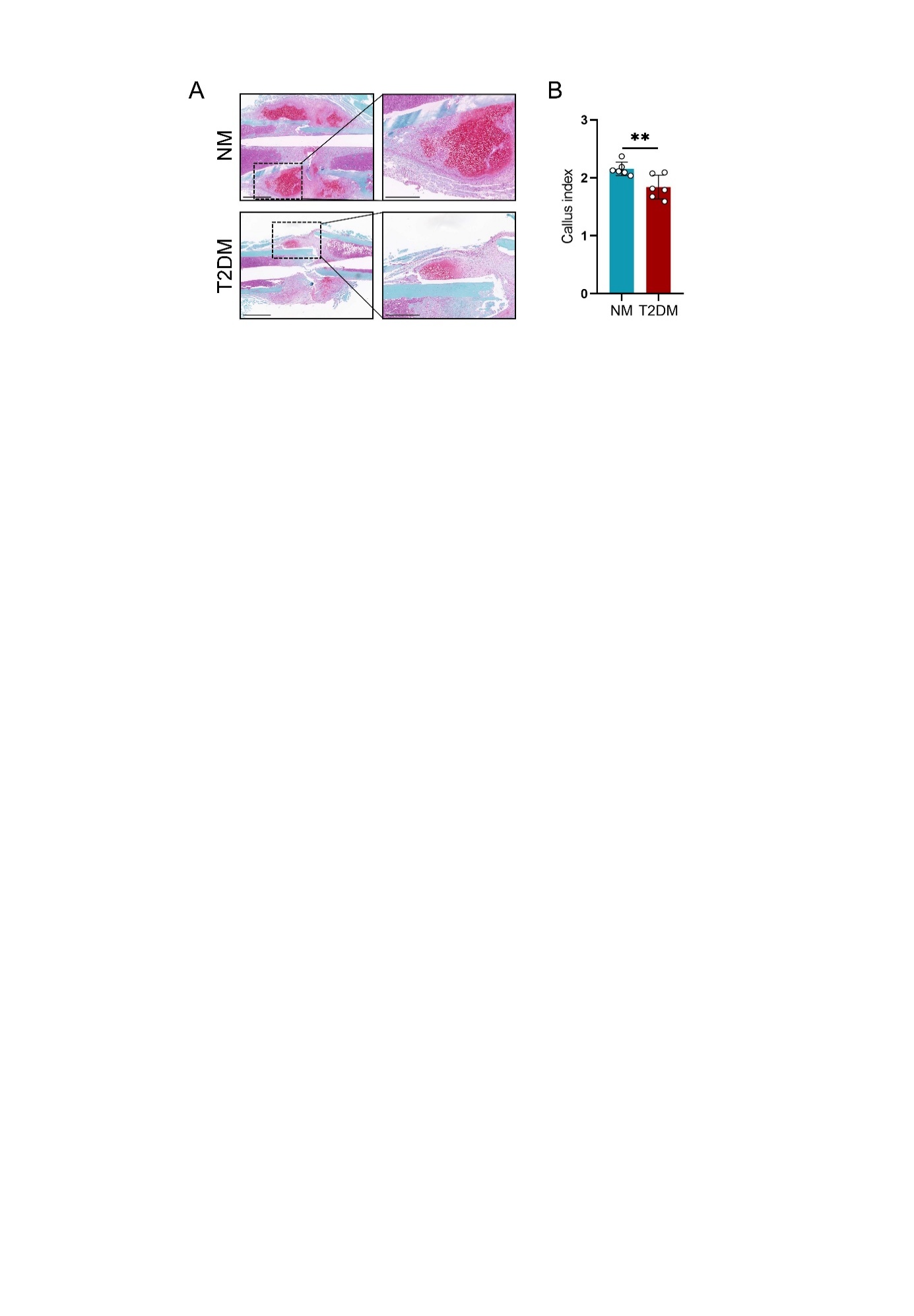


**Fig S2.** Fracture healing was impaired in T2DM mice. A) Representative images of safranin O staining and B) calculated callus index of callus at 10 days after fracture in NM and T2DM mice (*n* = 6) (Scale bars, 1 mm (left panels) and 0.5 mm (right panels)). Data are shown as mean ± SD; ***p*<0.01 by unpaired Student’s two-tailed t-test.


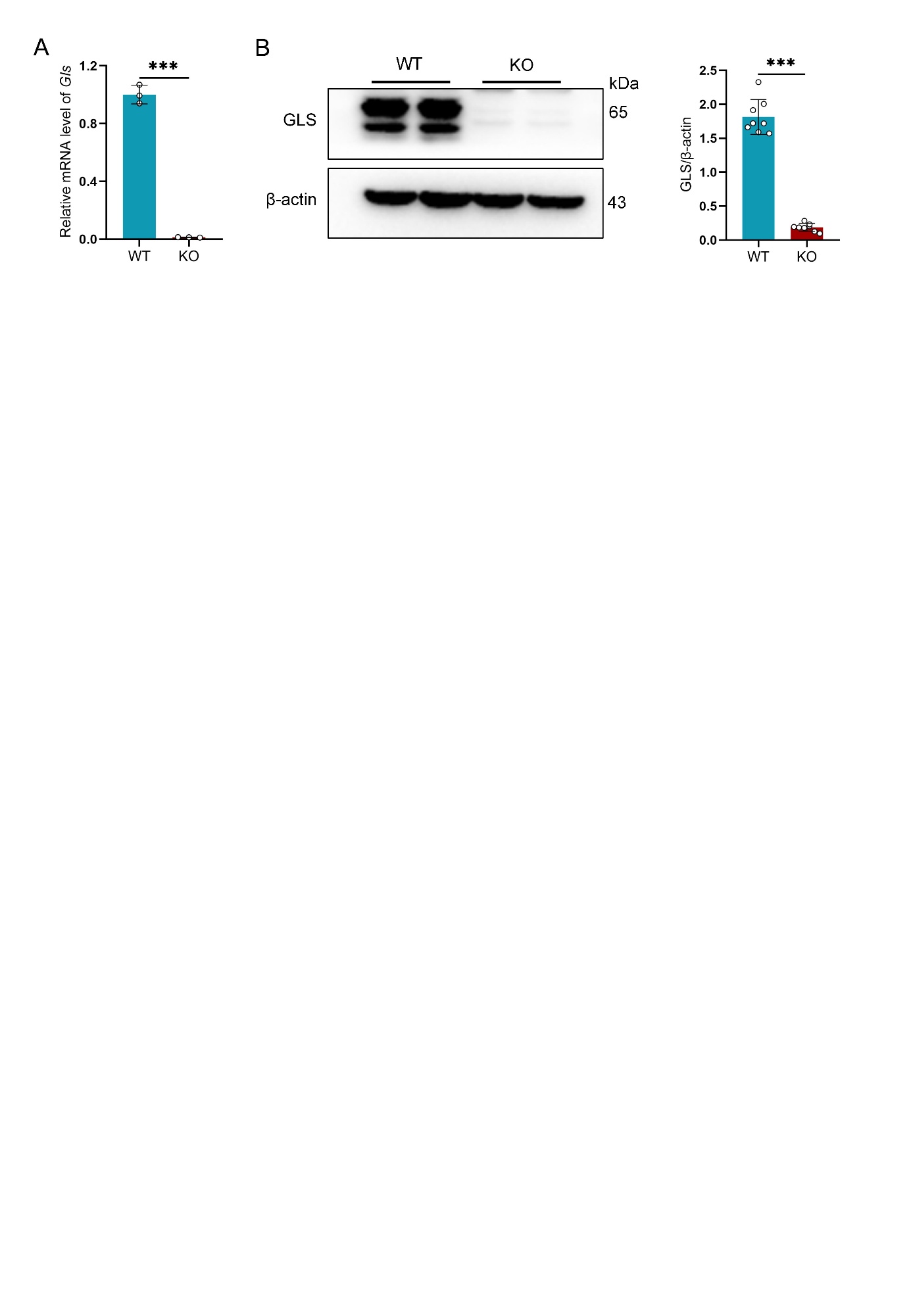


**Fig S3**. GLS expression was reduced in BMDMs isolated from *Gls^fl/fl^;Lyz2-Cre* mice.A) mRNA levels of *Gls* in BMDMs isolated from *Gls^fl/fl^* (WT) and *Gls^fl/fl^;Lyz2-Cre* (KO) mice (*n* = 3). B) Protein levels of GLS in BMDMs isolated from *Gls^fl/fl^* (WT) and *Gls^fl/fl^;Lyz2-Cre* (KO) mice (*n* = 8). Data are shown as mean ± SD; ****p*<0.001 by unpaired Student’s two-tailed t-test.


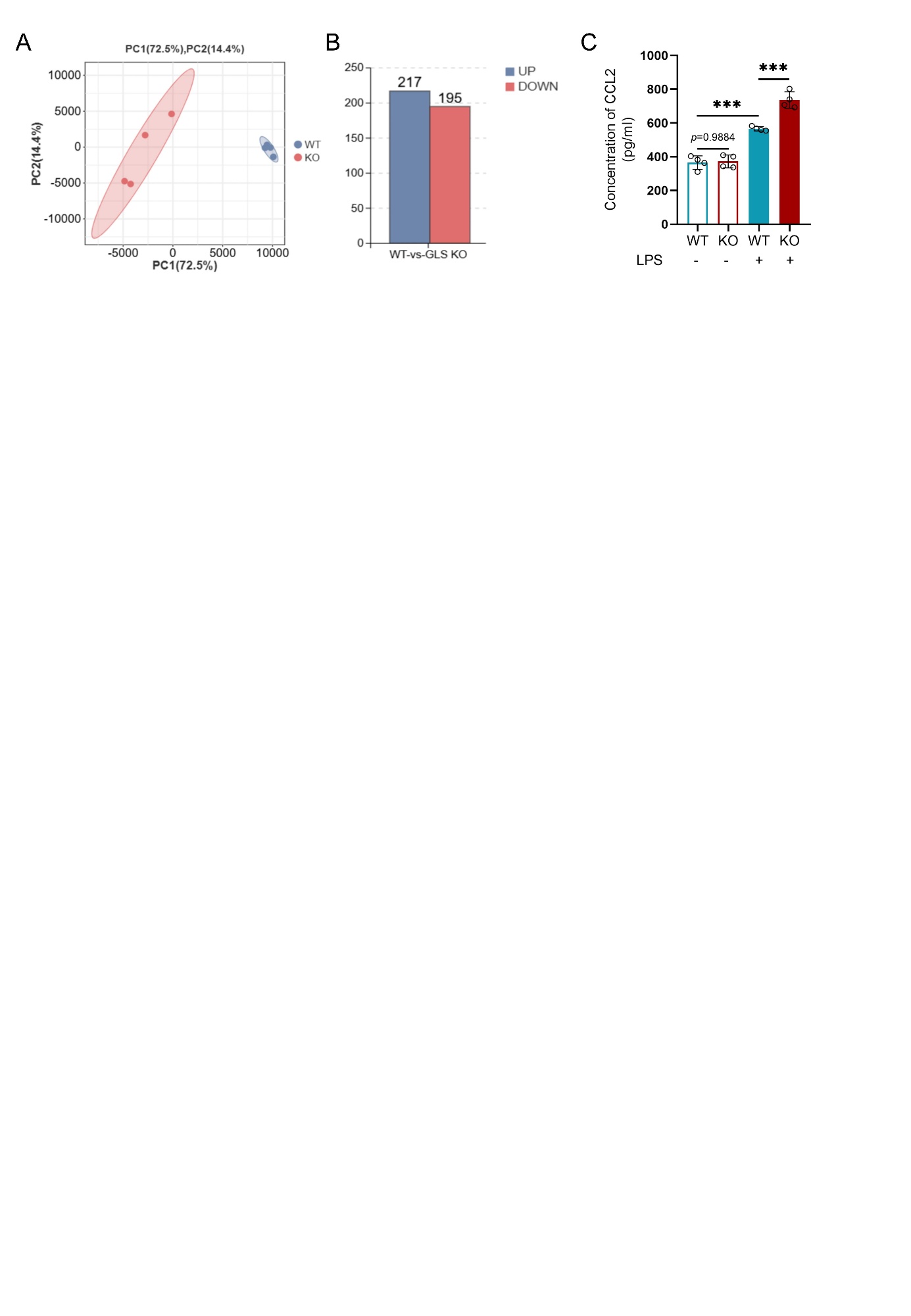


**Fig S4.** GLS deficiency altered macrophage phenotype and function. A) PCA and B) numbers of the differentially expressed genes in BMDMs isolated from *Gls^fl/fl^* (WT) and *Gls^fl/fl^;Lyz2-Cre* (KO) mice (*n* = 4). C) Concentrations of CCL2 in the supernatants of WT and KO BMDMs treated with vehicle or LPS for 6 h (*n* = 4). Data are presented as mean ± SD; ****p* < 0.001 by one-way ANOVA.


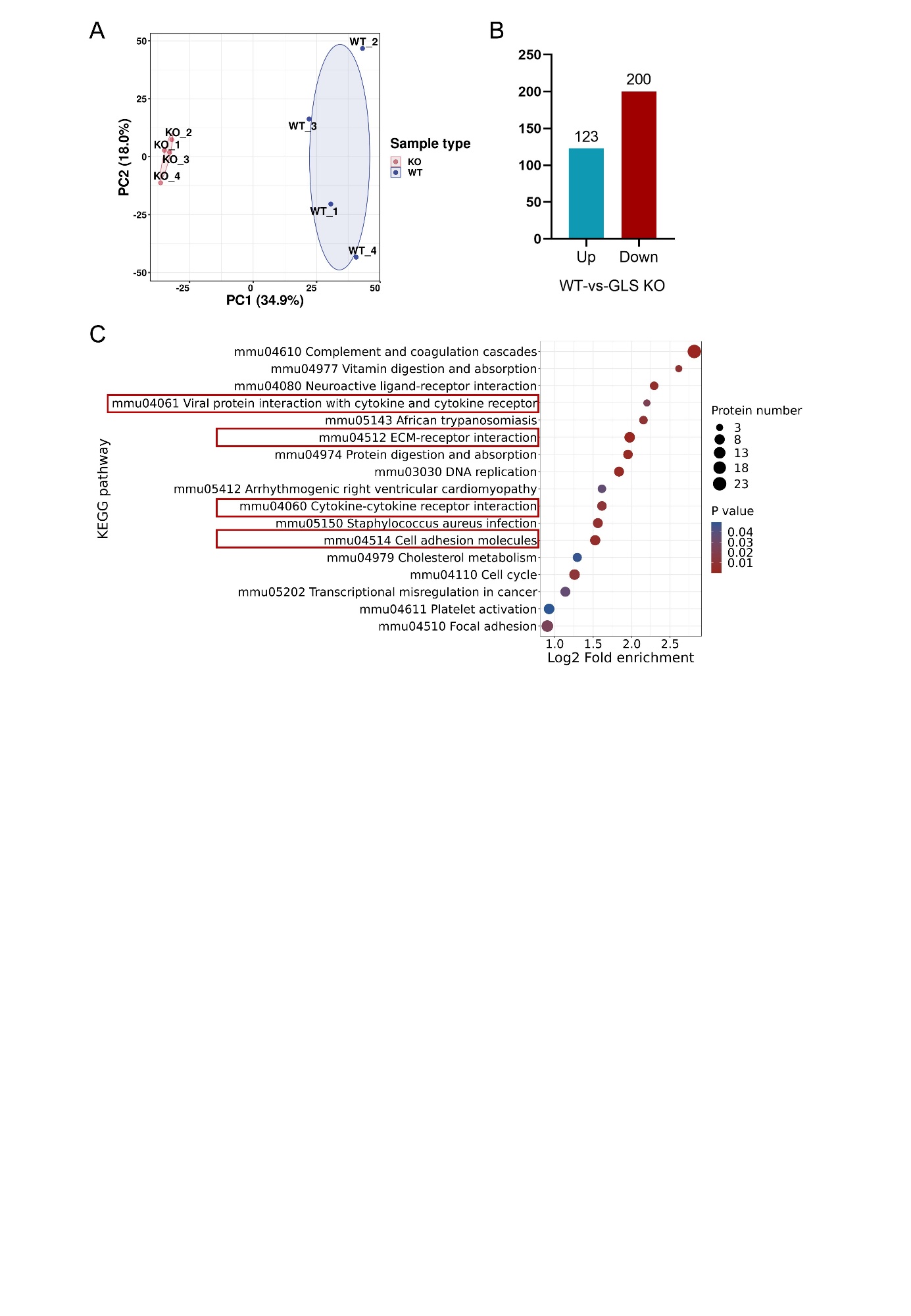


**Fig S5.** GLS deficiency affected macrophage cytokine secretion. A) PCA and B) numbers of the differentially secreted proteins in supernatants of BMDMs isolated from *Gls^fl/fl^* (WT) and *Gls^fl/fl^;Lyz2-Cre* (KO) mice (*n* = 4). C) KEGG pathway enrichment analysis revealing the top 20 pathways enriched in supernatants of WT and KO BMDMs.


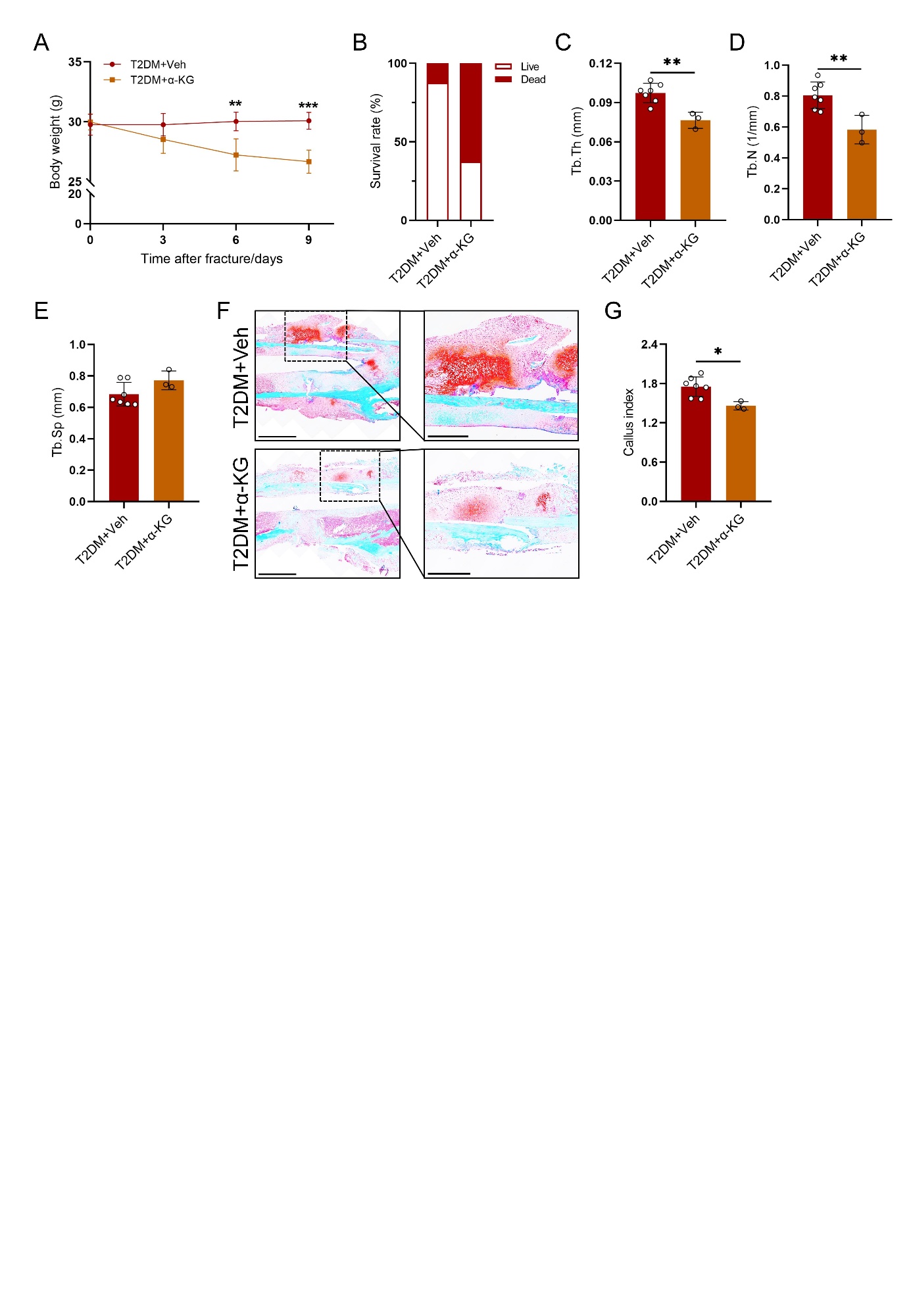


**Fig S6.** Systemic supplementation of α-KG had no beneficial effects on fracture healing but exacerbated survival of T2DM mice. A) Body weight of T2DM mice after fracture with or without α-KG supplementation (*n* = 8). B) Survival rate of T2DM mice after fracture with or without α-KG supplementation. C) Tb.Th, D) Tb.N and E) Tb.Sp of callus in T2DM mice treated with or without α-KG supplementation at 10 days after fracture (*n* = 7 in T2DM+Veh and *n* = 3 mice in T2DM+α-KG). F) Representative image and G) calculated callus index of Safranin O staining shows the formation of cartilage (Red) and bone (Green) in callus of T2DM mice treated with vehicle or α-KG at 10 days after fracture (*n* = 7 in T2DM+Veh and *n* = 3 mice in T2DM+α-KG) (Scale bars, 1 mm (left panels) and 0.5 mm (right panels)). Data are shown as mean ± SD. **p*<0.05 and ***p*<0.01 by unpaired Student’s two-tailed t-test.


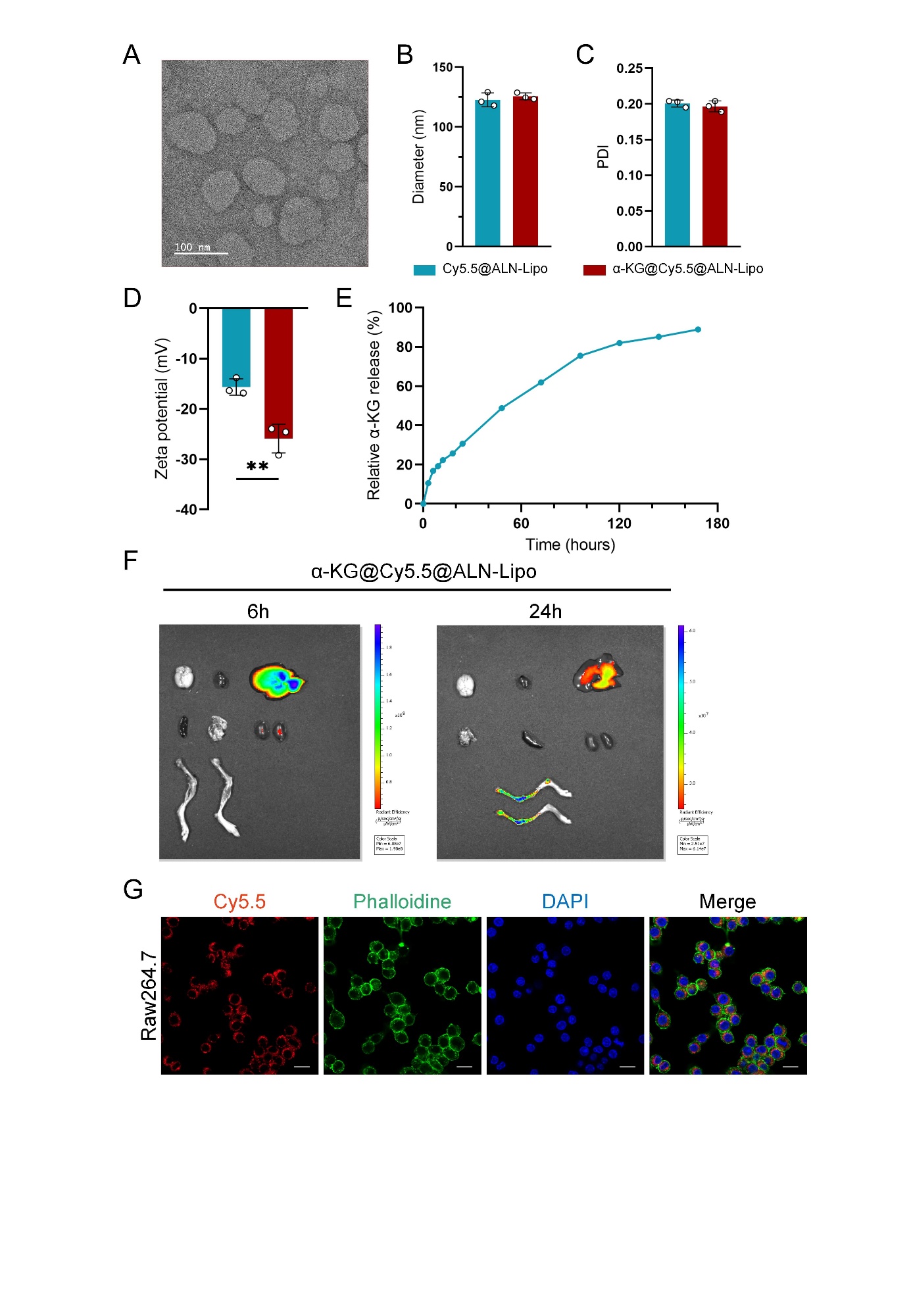


**Figure S7.** Design and characterization of α-KG@ALN-liposome. A) Representative TEM image of bone targeted liposome decorated with alendronate (ALN) containing α-KG and Cy5.5. B) Diameter, C) PDI and D) Zeta potential of Cy5.5@ALN-Lipo and α-KG@Cy5.5@ALN-Lipo (*n* = 3). E) Cumulative release of α-KG of α-KG@Cy5.5@ALN-Lipo when suspended in PBS for 7 days (*n* = 3). F) Representative fluorescence images in major organs including bones at 6 and 24h after injection of α-KG@Cy5.5@ALN-Lipo. G) Representative images of RAW264.7 treated with α-KG@Cy5.5@ALN-Lipo showing uptake of the liposome containing α-KG and Cy5.5 (Scale bars, 20 μm). Data are shown as mean ± SEM (B to D) and mean ± SD (E); ***p*<0.01 by unpaired Student’s two-tailed t-test.

**
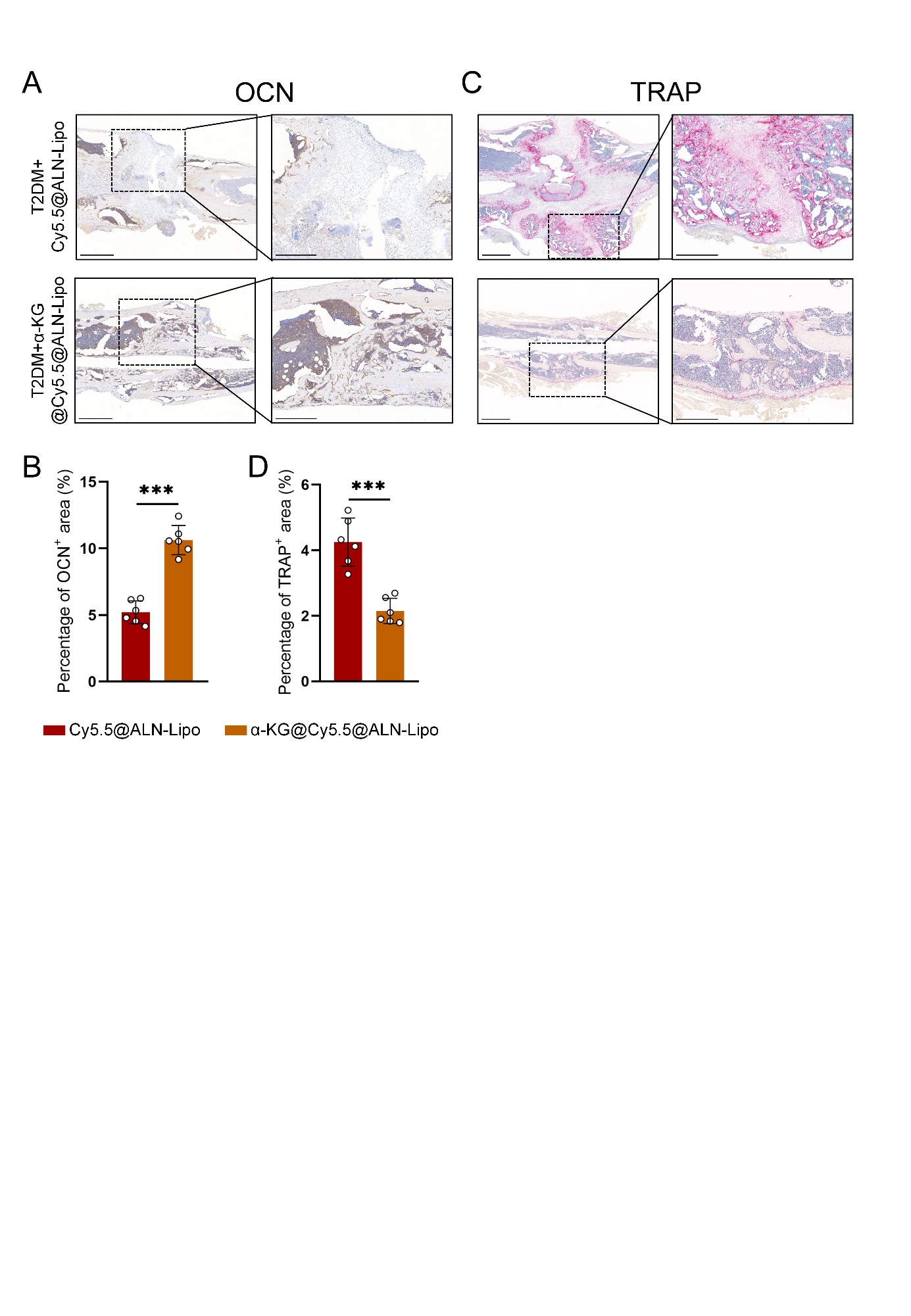
**

**Figure S8.** Diverseeffects of α-KG@Cy5.5@ALN-Lipo on osteoblasts and osteoclasts in fracture callus of T2DM mice. A) Representative images of immumohistochemical staining and B) quantitative analysis showing OCN^+^ osteoblasts in callus of T2DM mice treated with Cy5.5@ALN-Lipo or α-KG@Cy5.5@ALN-Lipo (*n* = 6). C) Representative images of TRAP staining and D) quantitative analysis showing TRAP^+^ osteoclasts in callus of T2DM mice treated with Cy5.5@ALN-Lipo or α-KG@Cy5.5@ALN-Lipo (*n* = 6). (Scale bars, 1 mm (left panels) and 0.5 mm (right panels)). Data are shown as mean ± SD; ****p*<0.001 by unpaired Student’s two-tailed t-test.


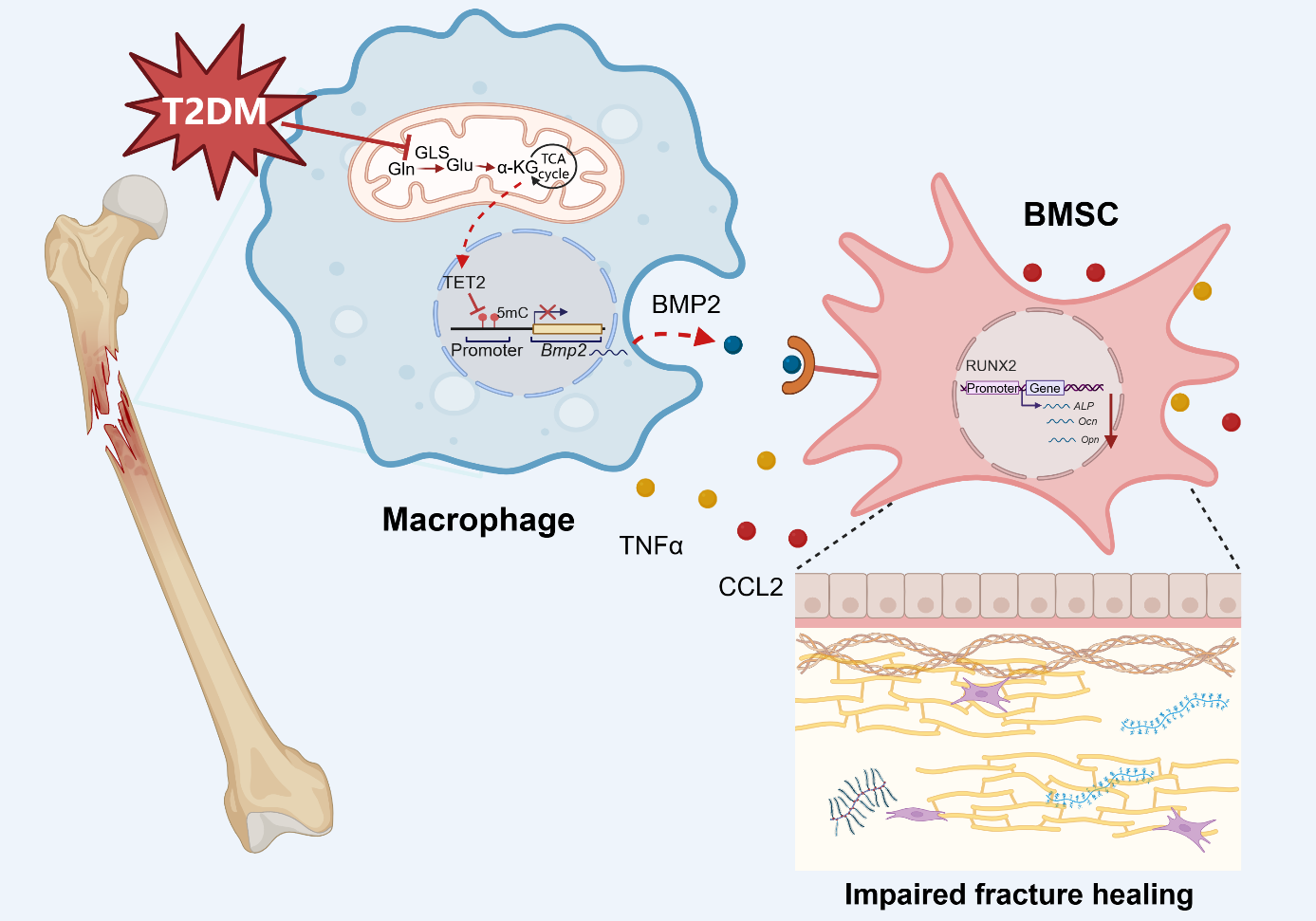


**Fig S9: Detailed mechanism for the inhibited macrophage glutaminolysis impaired fracture healing in type 2 diabetes mellitus.** The expression and activity of GLS in macrophages is inhibited under T2DM condition, reducing glutamate and α-KG levels. Inhibited glutaminolysis decreased α-KG level, which inhibited TET activity and increased cytosine methylation in the promoter region of *Bmp2*, thereby reduced its secretion. In addition, GLS deficiency aggravates inflammation in macrophages. Collectively, these changes hinder osteogenic differentiation of BMSCs and impaired fracture healing in T2DM.

**Supplemental Table 1.** Study population characteristics for participants in Figure 1K to P.

| Characteristics* | Healthy control  (*n* = 10) | T2DM  (*n* = 20) | P Value  (Healthy control vs T2DM) |
| --- | --- | --- | --- |
| Age [years] | 53.40 ± 10.56 | 57.70 ± 7.34 | 0.2027 |
| Female sex, n [%] | 4 (40.00) | 6 (30.00) | / |
| Body mass index [kg/m^2^] | 21.25 ± 2.07 | 24.45 ± 3.28 | 0.0091 |
| Fasting blood glucose [mM] | 5.10 ± 0.39 | 8.78 ± 2.21 | <0.0001 |
| Glycated hemoglobin (HbA1c) [%] | / | 8.45 ± 1.83 | / |

*Values presented are unadjusted means ± SD or numbers (percentages). P value was analyzed by unpaired Student’s two-tailed t-test.

**Supplemental Table 2.** Primer information.

| Targeted gene | species | Primer sequences (5’-3’) |
| --- | --- | --- |
| *GLS* | Human | TCTACAGGATTGCGAACGTCT(F)  CTTTGTCTAGCATGACACCATCT(R) |
| *36B4* | Human | AGATGCAGCAGATCCGCAT(F)  GTTCTTGCCCATCAGCACC(R) |
| *Gls* | Mouse | GCACTACACTTTGGACACCA(F)  TAGCAACCCGTCGAGATT(R) |
| *Alp* | Mouse | GTTGCCAAGCTGGGAAGAACAC(F)  CCCACCCCGCTATTCAAAC(R) |
| *Ocn* | Mouse | GAACAGACTCCGGCGCTA(F)  AGGGAGGATCAAGTCCCG(R) |
| *Opn* | Mouse | GACGATGATGACGACGACGATGAC(F)  GTGTGCTGGCAGTGAAGGACTC(R) |
| *Runx2* | Mouse | TTCTCCAACCCACGAATGCAC(F)  CAGGTACGTGTGGTAGTGAGT(R) |
| *Inos* | Mouse | ATCTTGGAGCGAGTTGTGGATTGTC(F)  TAGGTGAGGGCTTGGCTGAGTG(R) |
| *Tnfa* | Mouse | CGCTCTTCTGTCTACTGAACTTCGG(F)  GTGGTTTGTGAGTGTGAGGGTCTG(R) |
| *Il1b* | Mouse | CACTACAGGCTCCGAGATGAACAAC(F)  TGTCGTTGCTTGGTTCTCCTTGTAC(R) |
| *Il6* | Mouse | CTTCTTGGGACTGATGCTGGTGAC  TCTGTTGGGAGTGGTATCCTCTGTG |
| *Arg1* | Mouse | AGACAGCAGAGGAGGTGAAGAGTAC(F)  AAGGTAGTCAGTCCCTGGCTTATGG(R) |
| *Fizz1* | Mouse | TCCCTCCACTGTAACGAAGACTCTC(F)  GCCACAAGCACACCCAGTAGC(R) |
| *Mrc* | Mouse | GTCTGAGTGTACGCAGTGGTTGG(F)  TCTGATGATGGACTTCCTGGTAGCC(R) |
| *Il10* | Mouse | TGCCAAGCCTTATCGGAAATGATCC(F)  AGCCGCATCCTGAGGGTCTTC(R) |
| *β-actin* | Mouse | GCTGTGCTATGTTGCCCTAGACTTC(F)  GGAACCGCTCATTGCCGATAGTG(R) |
| *36B4* | Mouse | GAGACTGAGTACACCTTCCCAC(F)  ATGCAGATGGATCAGCCAGG(R) |
